# Supplementary material for: Zika viruses encode 5′ upstream open reading frames affecting infection of human brain cells
Source: Nat Commun. 2024 Oct 12;15:8822. doi: 10.1038/s41467-024-53085-9 (PMC11470053; doi:10.1038/s41467-024-53085-9)
Supplement: Supplementary file 1 — Supplementary Information [file 41467_2024_53085_MOESM1_ESM.pdf]

Supplementary Information

Supplementary Note 1:

Ribosomal frameshifting may produce an N-terminally truncated form of uORF2

As described in **Fig 2B** (**middle panel**), knockout of the uORF2 initiation codon or introduction of a premature termination codon (uORF2-PTC1) depressed uORF2 translation only two-fold in comparison to the American WT. In a further attempt to block the synthesis of the uORF2 product, a premature termination codon was placed within the middle of the uORF2 sequence (uORF2-PTC34, **Supp Fig 12A** in bright green) in the context of the uORF2-2A-FFLuc mRNA, and luciferase assays performed in transfected cells. As shown in **Supp Fig 12B**, introduction of PTC34 reduced uORF2 translation ~10-fold, suggesting that a signal for alternative uORF2 expression is present between residue 6 (PTC1) and residue 34 (PTC34). To investigate this, we looked for potential initiation sites between codons 6 and 34. No AUG codons are present, but four potential sites of non-AUG translation initiation could be accessed through leaky scanning of 40S initiation complexes on the mRNA. These amino acids were sequentially mutated (Mut1-AUC, Mut2-AGG, Mut3-AUU and Mut4-ACG; **Supp Fig 12A**) within the uORF2-2A-FFLuc reporter mRNA and tested in transfected cells (**Supp Fig 12C**). In all cases, luciferase levels were essentially unchanged, arguing against a role for leaky scanning in uORF2 expression.

We noticed that the sequence immediately downstream of the main ORF AUG has two A-rich stretches upstream of a stem-loop (cHP) that could potentially act as signals for programmed ribosomal frameshifting (PRF[1]), diverting a proportion of ribosomes translating the main ORF into the overlapping –1 (uORF2) reading frame (denoted FS2 and FS3 in **Supp Fig 12D**). Reducing the homopolymeric nature of each of these stretches by the introduction of two G residues within the uORF2-2A-FFLuc reporter reduced uORF2 translation at the FS3 site by

50% suggesting that at least half of uORF2 expression is derived from ribosomes that undergo –1 PRF very early during main ORF translation **(Supp Fig 12E)**. Disruption of the main ORF initiation codon (FS1) also significantly reduced uORF2 expression, consistent with a requirement for translation of the main ORF in uORF2 expression and frameshifting into the –1 frame at the 'blue' slippery sequence (FS3 region). Furthermore, within the context of the uORF2-PTC1-2A-FFLuc reporter mRNA, both FS1 and FS3 mutations reduced uORF2 expression further, presumably as ribosomes can no longer access uORF2 following initiation at the main ORF AUG codon (FS1) and frameshifting into uORF2 at the FS3 signal (**Supp Fig 12F**).

The dual mode of uORF2 expression, via initiation from the UUG initiation codon, and also expression of an N-terminally truncated version by –1 ribosomal frameshifting of ribosomes initiating at the main polyprotein (0) frame is an unexpected finding. Based on previous work[1], a likely site of frameshifting in this region is the C_CCA_AAA putative "slippery" sequence located four codons into the polyprotein and three nucleotides upstream of the cHP stem-loop. Consistent with this hypothesis, mutation of this sequence to non-slippery C_CCG_AAG reduced uORF2 expression in reporter gene assays (**Supp Fig 12E**). Frameshifting to CCC_AAA (uORF2 frame) generates an N-terminal variant of uORF2, in which five amino acids derived from the start of the polyprotein replace the 14 N-terminal residues from initiation at the UUG codon. Conceivably, 'non-canonical' expression of uORF2 could also arise as a result of transcriptional slippage of T7 RNA-polymerase on the A-rich tract in this region[2], and indeed, transcriptional slippage has been reported to account for the proposed frameshifting that takes place at a similar position in the hepatitis C virus (HCV) genome[3]. However, analysis of the ZIKV RNA-Seq data does not reveal any heterogeneity in this A-rich region, in contrast to what was observed with HCV[3].

Supplementary Figure Legends

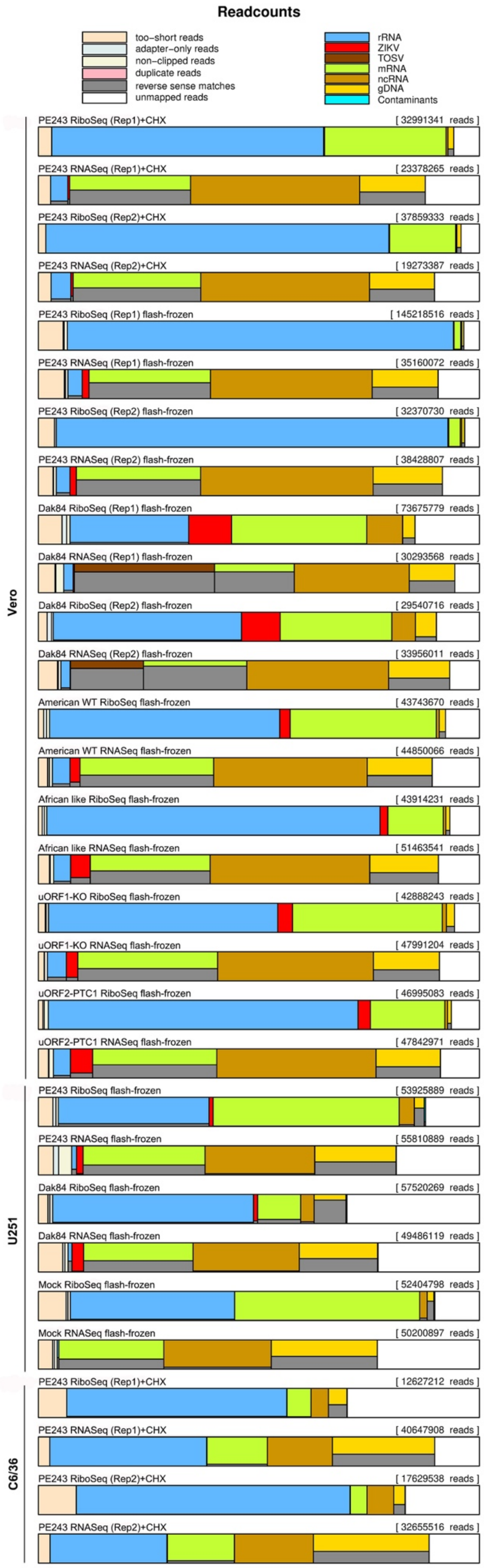

Supp. Figure 1. Composition of libraries. Reads were mapped to ZIKV RNA, Toscana virus (TOSV) RNA, host rRNA, mRNA, ncRNA and gDNA databases, and our contaminant database. Reads mapping to gDNA are expected to derive from unannotated transcripts not present in the mRNA or ncRNA databases, but, since the direction of transcription is not annotated in the gDNA database, such reads constitute a mixture of forward and reverse-sense matches. Reverse-sense rRNA matches in the RNA-Seq samples are expected to derive from rRNA depletion kits which contain complementary sequences to rRNA.

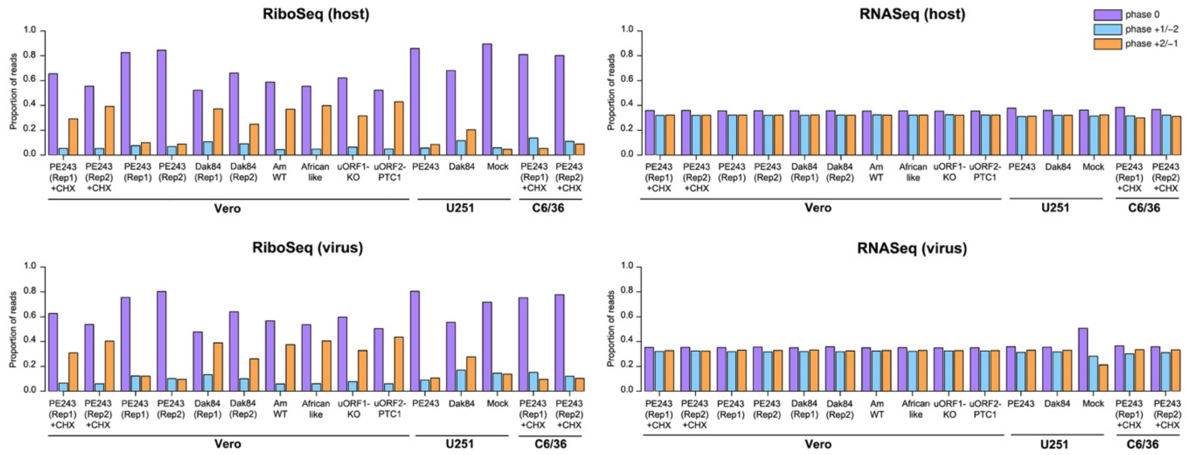

Supp. Figure 2. Phasing of reads. Proportion of reads (all read lengths) attributed to each phase, for positive-sense reads mapping within host CDSs (upper) and virus (lower). Phases correspond to which position within the codon the 5′ end of the read maps to (0: purple, 1: blue, 2: orange). The 5′ end coordinate of Ribo-Seq reads is influenced by the position of the translating ribosome, leading to a clear dominance of the 0 phase. For RNA-Seq reads, the 5′ end coordinate is determined by alkaline hydrolysis, so it does not result in a dominant phase. Note that the low virus read count for mock samples in the virus plots (lower) makes the phase distributions prone to noise.

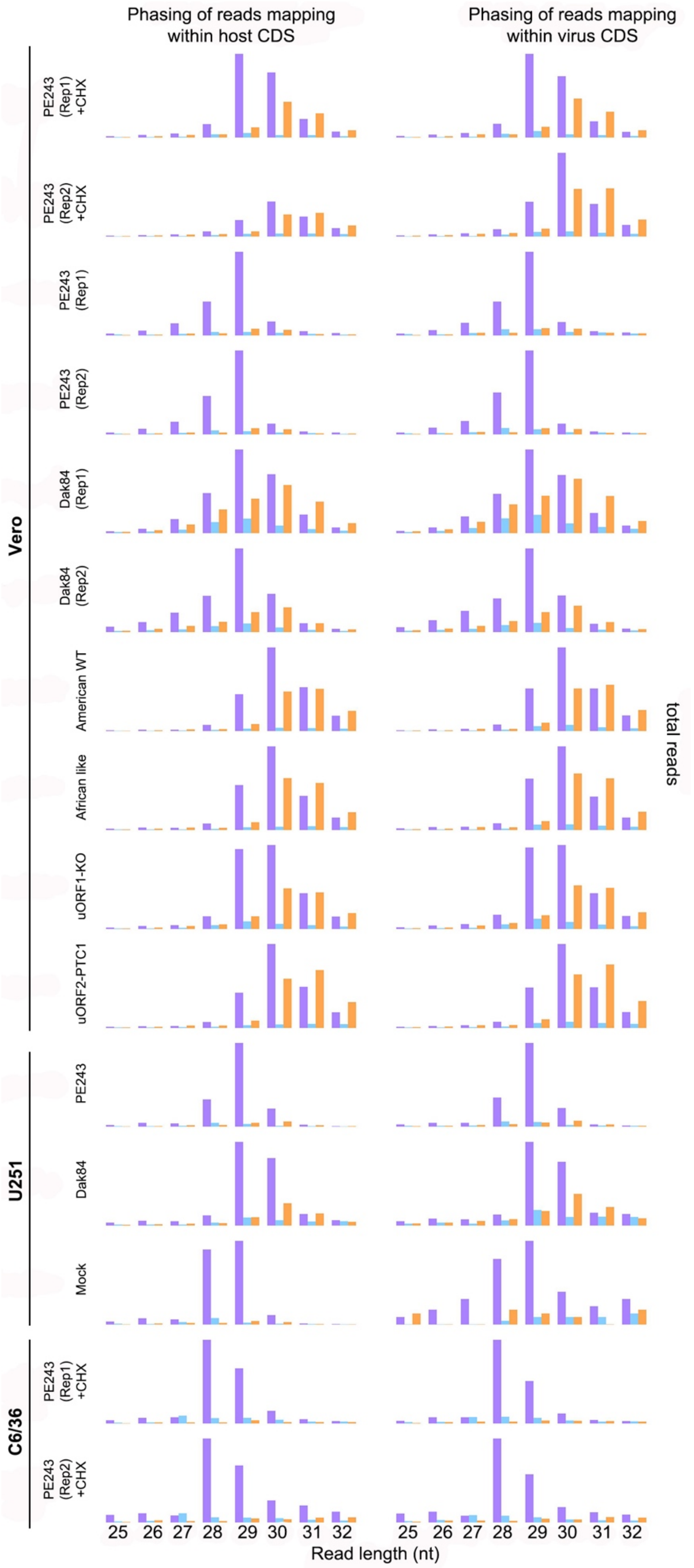

Supp. Figure 3. Phasing of reads distributed by read length (from 25 to 32 nt). Phasing shown as in **Supp Fig 2**.

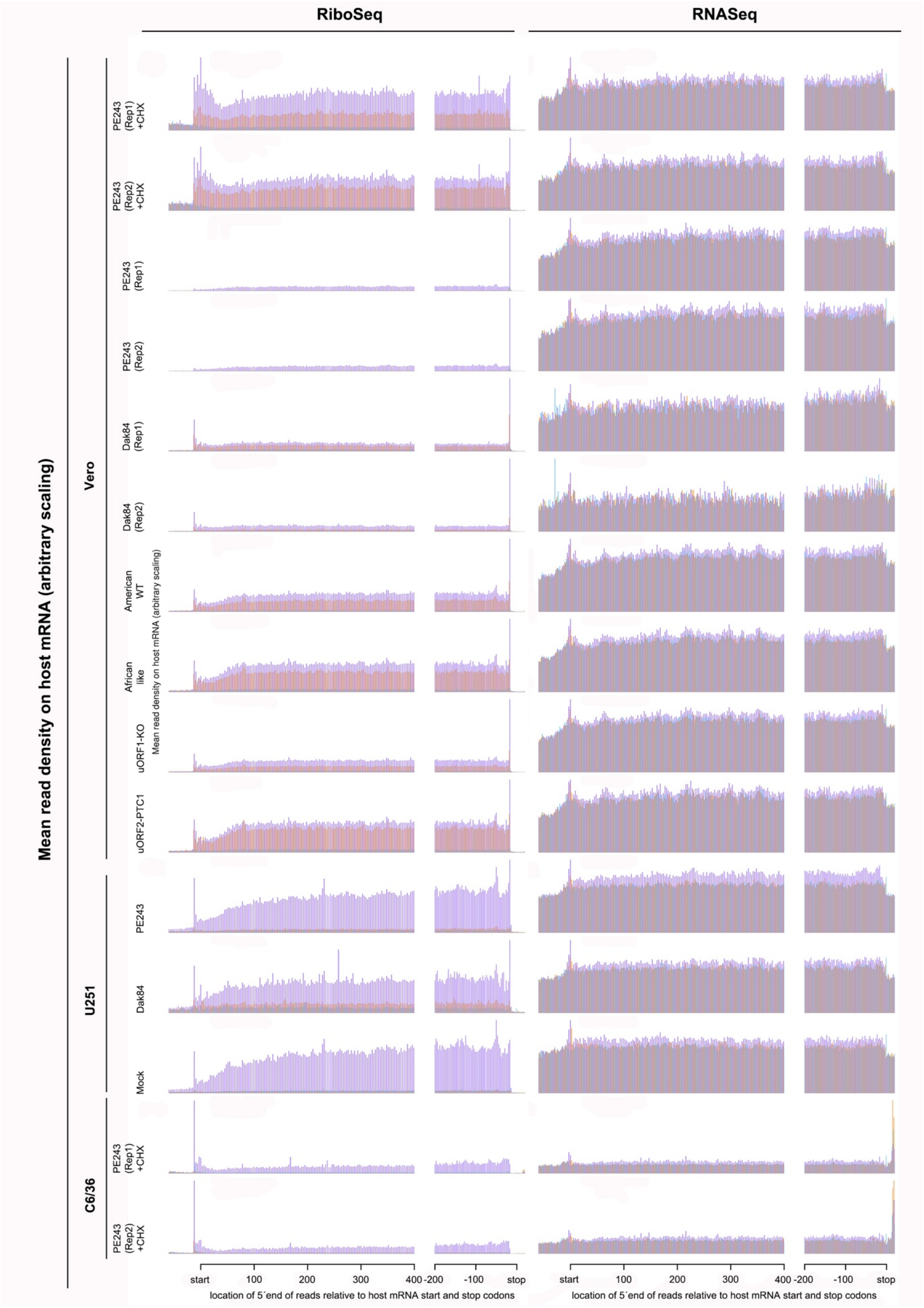

Supp. Figure 4. RPF and RNA-Seq distributions on host mRNAs. Histograms of RPF (left) and RNA-Seq read (right) 5′ end positions relative to annotated initiation and termination

codons summed over all host RefSeq mRNAs for the Ribo-Seq (left) and RNA-Seq (right) libraries. To account for different library sizes, histograms are normalised by positive-sense host mRNAs.

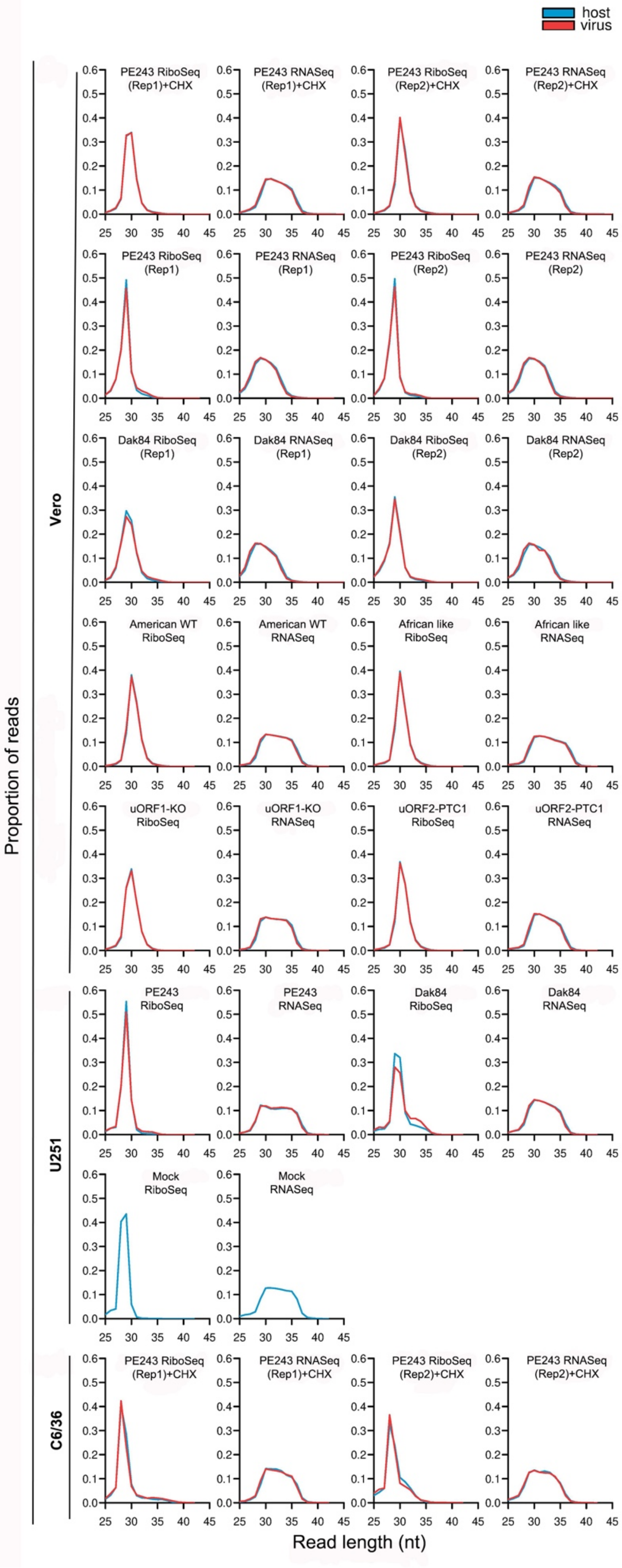

Supp. Figure 5. Comparison of read length distributions for virus and host mRNA. Length distributions for reads mapping to positive-sense host mRNAs (blue) and virus RNA (red). Every panel shows the distribution of the number of reads per read length in each library to facilitate the comparison of distribution shapes. Differences between host and virus distributions are indicative of contamination.

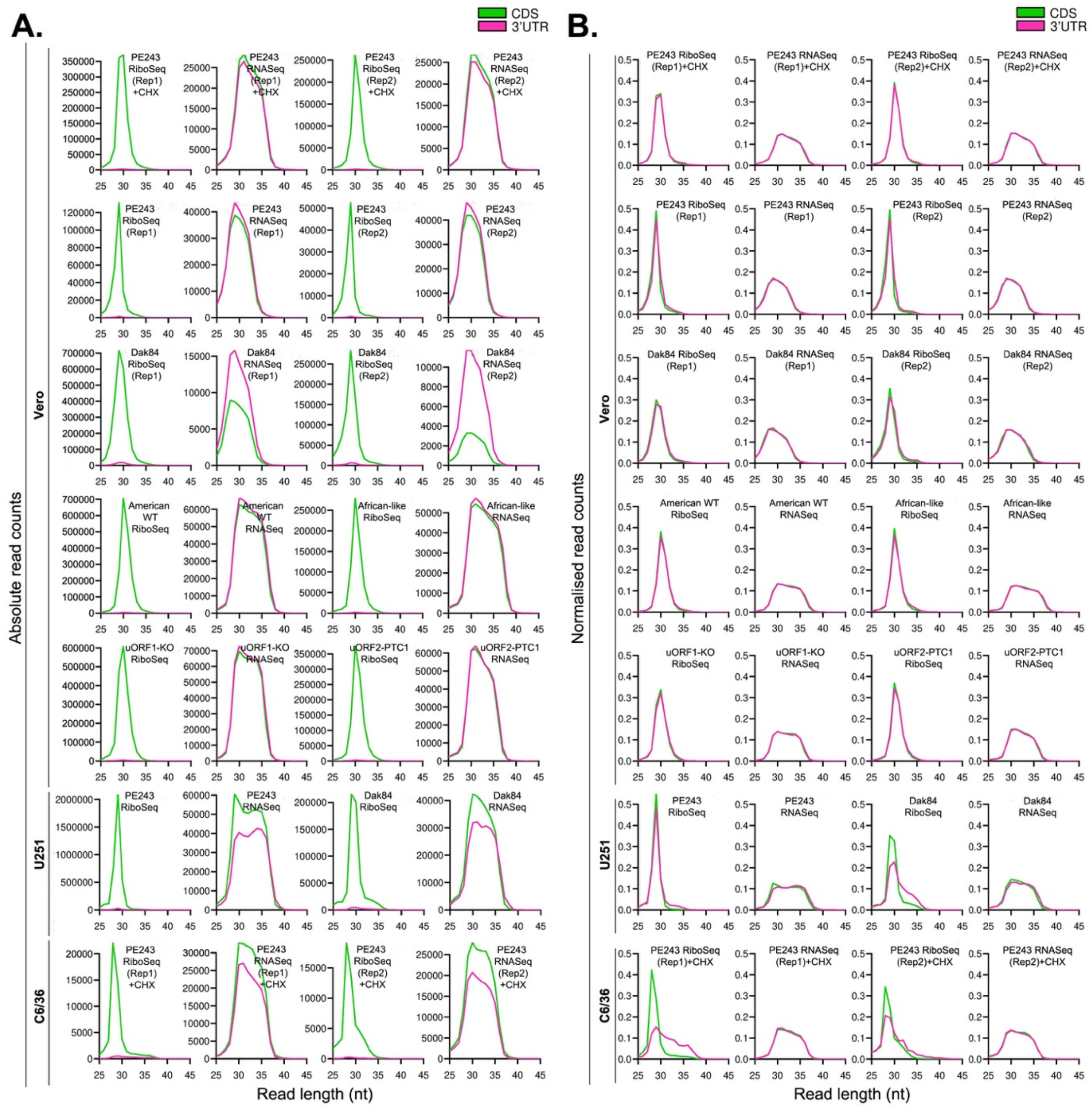

Supp. Figure 6. Comparison of the length distribution of reads mapping to host mRNA coding regions and 3′ UTRs. Reads were counted in windows from 10 to 100 codons upstream (CDS; green) or downstream (3′ UTR; magenta) of annotated termination codons, and summed over all host mRNAs. (**A**) Each panel shows the absolute read counts, allowing comparison of the CDS and 3′ UTR read densities. For all Ribo-Seq samples, 3′ UTR occupancy is very low compared to CDS occupancy, whereas, for RNA-Seq, 3′ UTR occupancy is typically around 80% of CDS occupancy (this RNA-Seq value is less than 100% due to differences in the transcript isoforms present in the sample compared to the RefSeq mRNA database). (**B**) Each

panel shows the distributions normalized to have equal total sums so that the shapes of the CDS and 3′ UTR distributions can be compared. For RNA-Seq, the two distributions have essentially identical shapes. For Ribo-Seq, differences in the two distributions provide an indicator of the level of non-RPF contamination present in the sample[4].

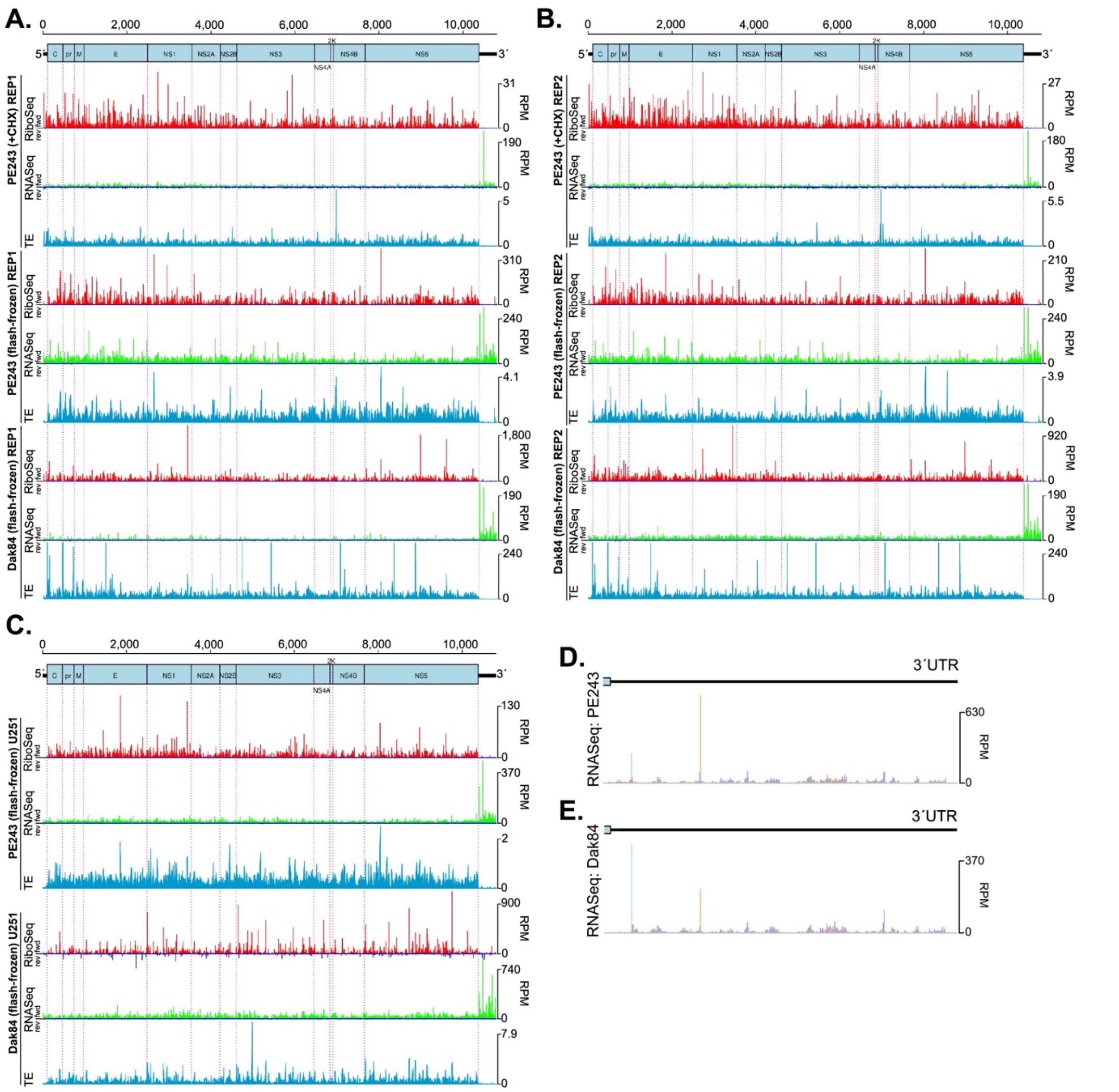

Supp. Figure 7. ZIKV RNA synthesis and translation. (**A**) Ribo-Seq (red) and RNA-Seq (green) densities in reads per million mapped reads (RPM) of repeat 1 (REP1) of American isolate PE243 (MOI:3) at 24 h p.i. in Vero cells pre-treated with CHX (upper panel), flash-frozen Vero cells (middle panel); or flash-frozen Vero cells infected with African isolate Dak84 (MOI:3) at 24 h p.i. (lower panel) as described in **Fig 1**. TE (light blue) is translational efficiency. Vero cells pre-treated with CHX panel is the non-truncated version of **Fig 1B**. (**B**) Repeat 2 (REP2) as in **A**. (**C**) Flash-frozen U251 cells infected with PE243 (MOI:3, upper panel) or Dak84 (MOI:3, lower panel) at 24 h p.i. (**D-E**) Zoom plots of RNA-Seq densities of replicate 1 of flash-frozen Vero cells at 24 h p.i. in the PE243 (**D**) or Dak84 (**E**) 3′ UTR.

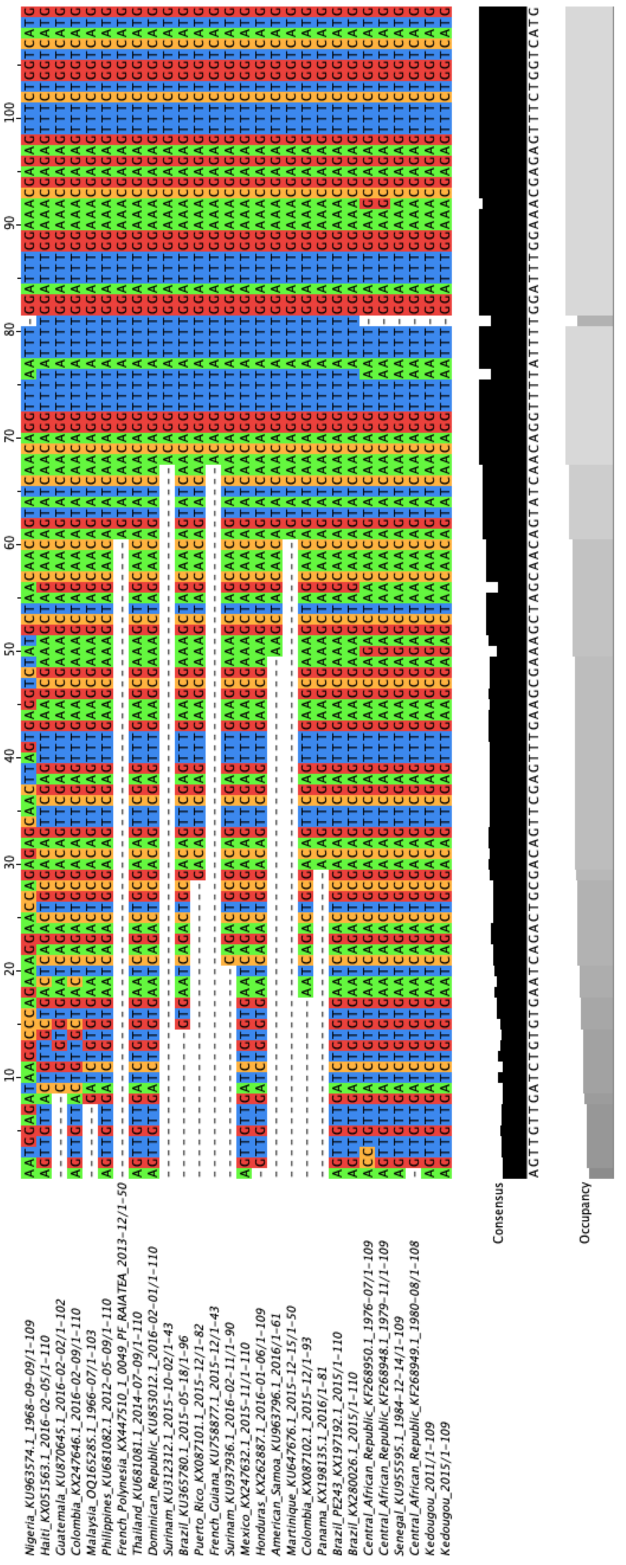

Supp Figure 8. Alignment of representative 5′ UTR sequences of African and Asian/American isolates. This alignment includes seven African isolates from 1968 to 2015 and 21 Asian/American isolates from 1966 to 2016. GenBank accession numbers for the sequences used are included in the name. Multiple sequence alignment was generated using Clustal Omega (EMBL-EBI), and the figure was generated using Jalview 2.11.3.2.

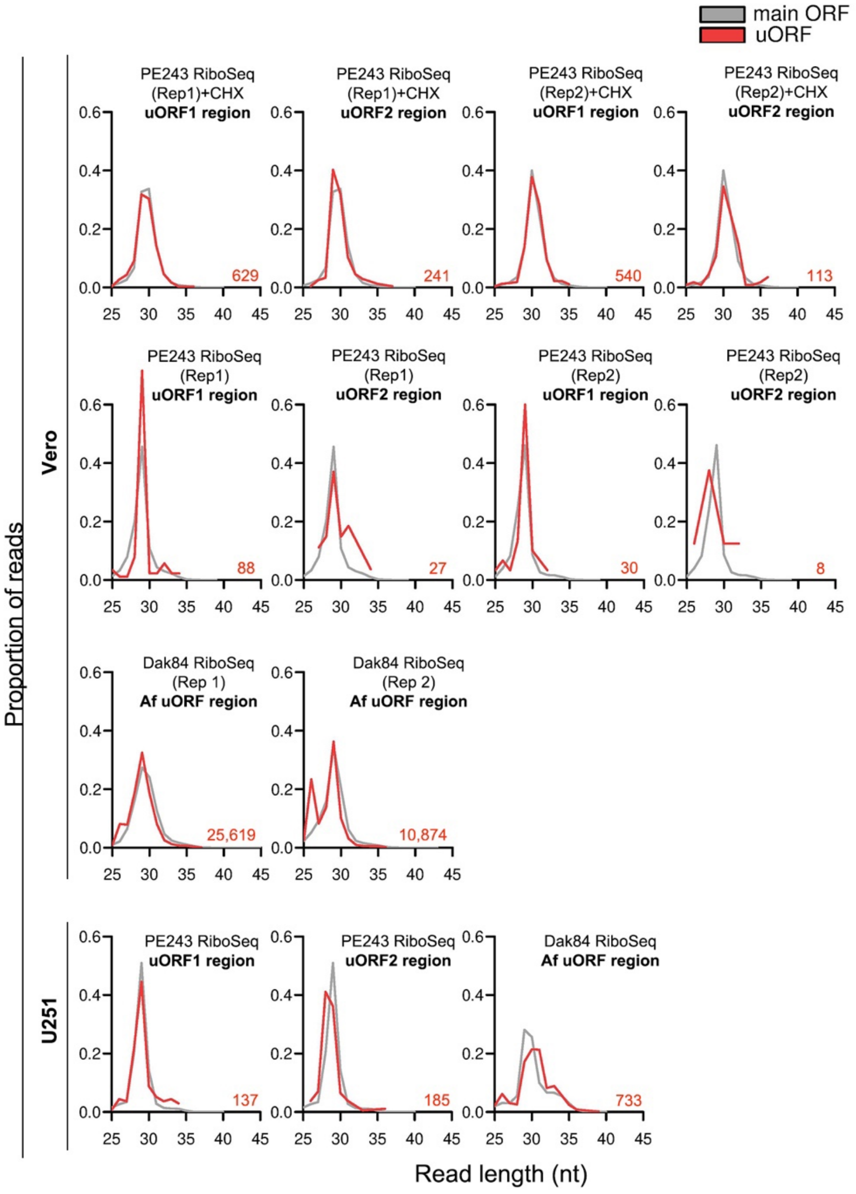

Supp. Figure 9. Length distribution of Ribo-Seq reads mapping to the different uORFs (region 25-79 nt for uORF1, region 80-107 nt for uORF2 and region 25-106 for African uORF, in red) **and Ribo-Seq reads mapping to the whole polyprotein** (in grey) for Vero and U251 cells infected with American isolate PE243 and African isolate Dak84. In red is also indicated the number of reads mapping to that specific region.

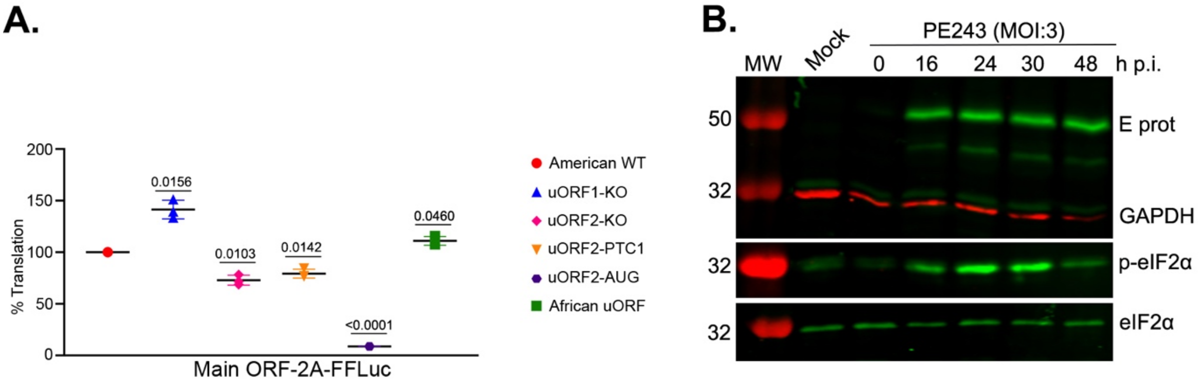

Supp. Figure 10. Analysis of ZIKV uORF translation using luciferase reporters. (**A**) Relative FF-Luc/Ren-Luc activity of main-ORF-2A-FFLuc mutant reporters in U251 cells. Cells were harvested at 30 h p.t.. Experiments were performed in triplicate with three biological replicates. All *t*-tests were two-tailed and did not assume equal variance for the two populations being compared. All *p*-values are from comparisons of the mutant with the wild-type. (**B**) Western blot analysis of Vero cells infected with PE243 (MOI:3) and harvested at 0, 16, 24, 30 and 48 h p.i. Cell lysates were probed against viral E protein (E prot) and the phosphorylated version of eIF2α. GAPDH and eIF2α are used as loading controls. Molecular masses (in kDa) are indicated on the left. Experiments were performed in triplicate. Source data are provided as a Source Data file.

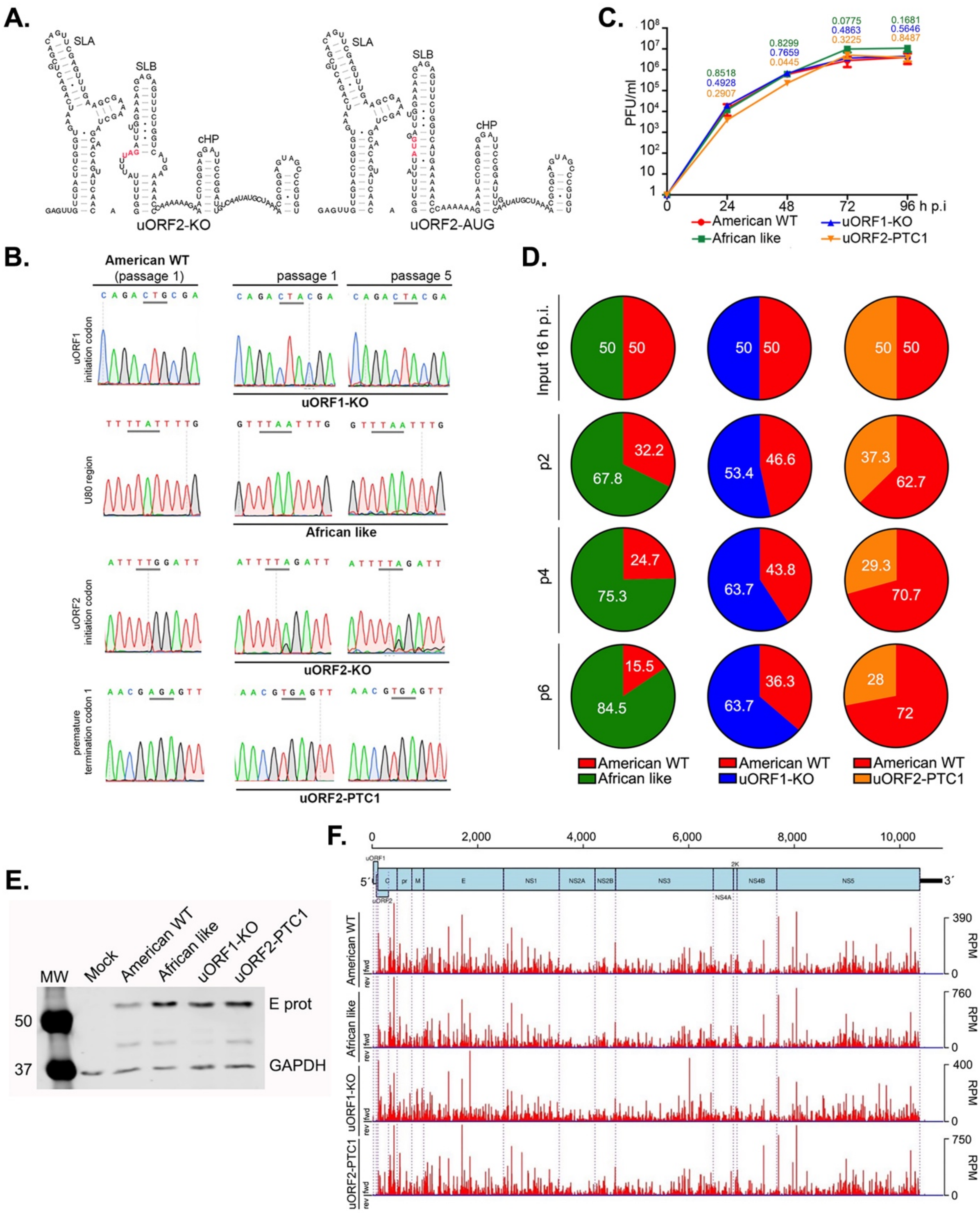

Supp. Figure 11. The significance of uORFs translation in virus infection. (**A**) SHAPE RNA secondary structure of the 5′ region (first 180 nucleotides) of the uORF2-KO and uORF2-AUG mutant viruses. Mutated codons are indicated in red. SLA (stem-loop A), SLB (stem-loop B) and cHP (capsid hairpin). (**B**) Sequencing histograms of RT-PCR products of the American WT, uORF1-KO, African-like, uORF2-KO and uORF2-PTC1 viruses at passage 1

and passage 5. Mutated nucleotides are underlined. (**C**) Time-course of Vero cells infected with ZIKV mutant viruses (MOI 0.01) for 96 h. Plaque assays were performed as described in **Fig 4C**. All *t*-tests were two-tailed and did not assume equal variance for the two populations being compared. All *p*-values (colour-coded) are from comparisons of the mutant virus with the American WT. (**D**) Pie charts of the competition assays of American WT and mutant viruses at 50:50 proportion in Vero cells as described in **Fig 4D**. Experiments were repeated independently eight times (raw data in **Supp Table 6**). (**E**) Western blot analysis of Vero cells infected with the American WT, African-like, uORF1-KO and uORF2-PTC1 viruses (MOI:3) for 24 h. Cellular extracts were probed against viral E protein and GAPDH (as loading control). Molecular masses (kDa) are indicated on the left. (**F**) Ribo-Seq density, in reads per million mapped reads (RPM), at 24 h p.i. in flash-frozen Vero cells infected with American WT, African-like, uORF1-KO and uORF2-PTC1 viruses (MOI:3) as described in **Fig 1B**. Source data are provided as a Source Data file.

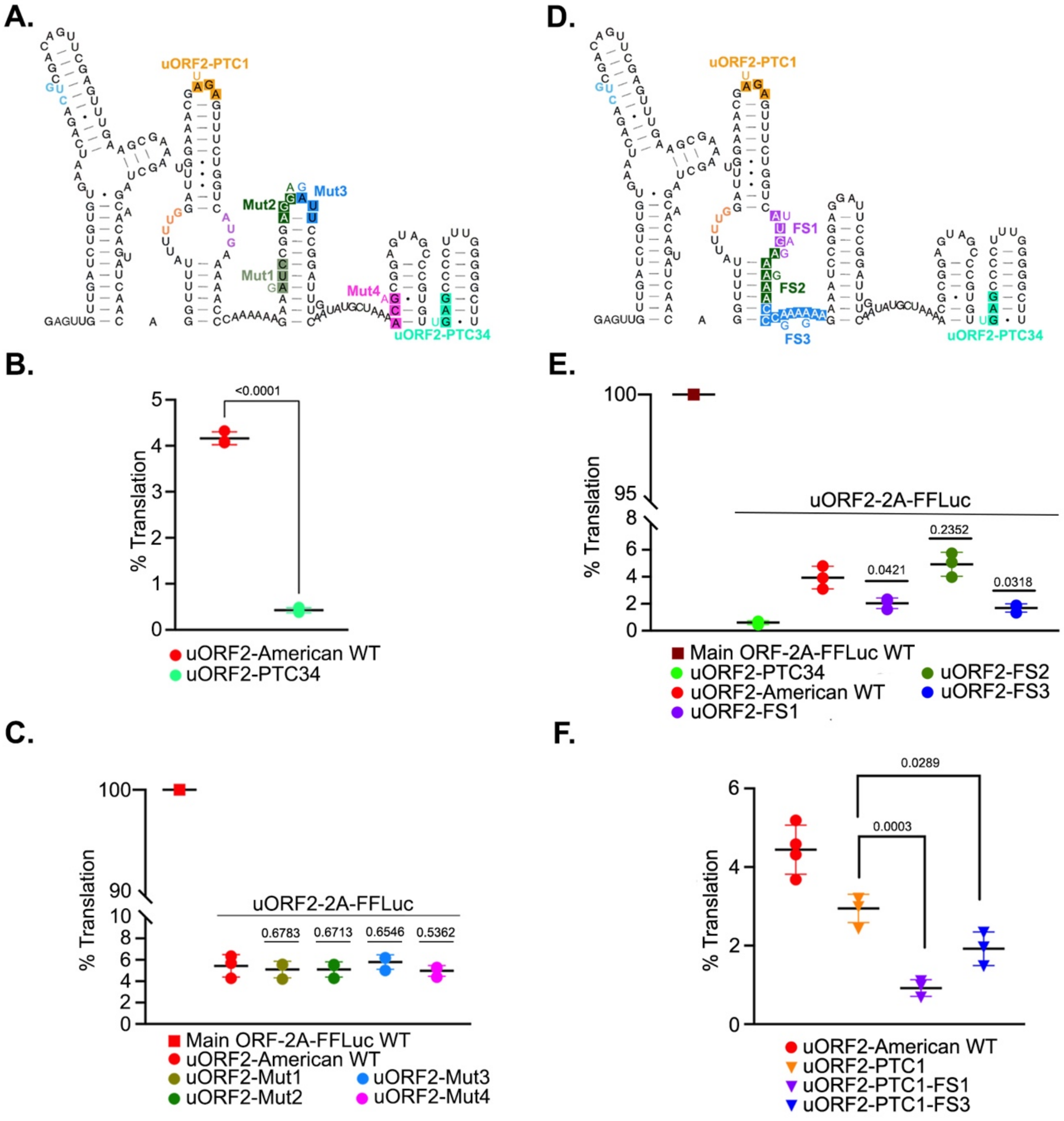

Supp Figure 12. Alternative mechanisms of uORF2 translation (**A**) Scheme of the 5′ region of the American WT. Premature termination codons (PTC) are colour-squared in orange and bright green for uORF2-PTC1 and uORF2-PTC34, respectively. Nucleotides corresponding to ‘alternative’ non-canonical initiation codons for uORF2 are colour-coded and named (Mut1-Mut4). Mutated nucleotides are located next to the original. (**B**) Relative FF-Luc activity for uORF2-PTC34 in Vero-transfected cells as in **Fig 2B**. (**C**) Relative FF-Luc activity for the different ‘alternative’ non-canonical initiation codon mutants of uORF2-2A-FFLuc in Vero-transfected cells. 100% translation accounted for the main ORF WT (red square). (**D**) Scheme

of the 5′ region of the American WT. The nucleotides corresponding to the main ORF initiation codon (FS1) and two potential slippery sequences (FS2 and FS3) are coloured in purple, green and blue, respectively. Mutated nucleotides are colour-coded and located adjacent to the original one. Relative FF-Luc activity of different mutants (PTC and FS) of uORF2-2A-FFLuc (**E**) and uORF2-PTC1-2A-FFLuc (**F**) in Vero-transfected cells as in **Fig 3B**. 100% translation accounted for the main ORF WT (brown square). All *t*-tests were two-tailed and did not assume equal variance for the two populations being compared. Experiments were performed in triplicate with three biological replicates. Source data are provided as a Source Data file.

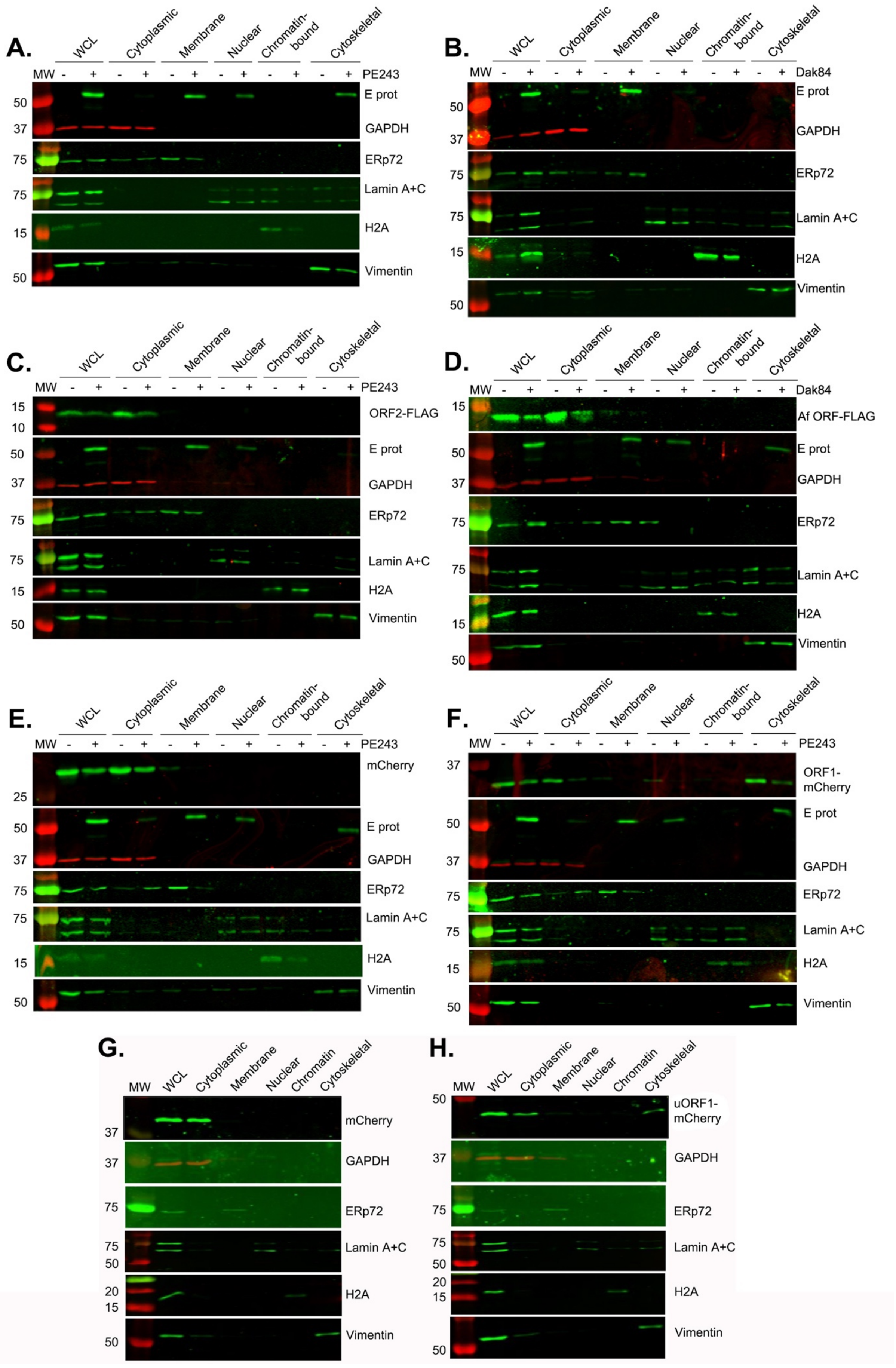
A.
WCL
Cytoplasmic
Membrane
Nuclear
Chromatin-bound
Cytoskeletal
MW
PE243
50
37
75
75
15
50
E prot
GAPDH
ERp72
Lamin A+C
H2A
Vimentin
B.
Dak84
C.
ORF2-FLAG
10
D.
Af ORF-FLAG
E.
mCherry
25
F.
ORF1-mCherry
G.
Chromatin
20
H.
uORF1-mCherry

Supp. Figure 13. Functional characterisation of ZIKV uORF-encoded proteins. (**A-F**) Vero cells were transfected with the corresponding pCAG plasmid for 24 hours and infected with either PE243 (American isolate) or Dak84 (African isolate) (MOI:1). Cells were harvested at 14 h p.i. (Dak84 infection) or 16 h p.i. (PE243 infection) and subjected to subcellular fractionation. Western blot analysis of total extract (WCL), cytoplasmic, membrane, nuclear, chromatin and cytoskeletal fractions was carried out as follows: membranes were probed with antibodies against FLAG or mCherry for detecting the *in trans* tagged protein; E protein (ZIKV infection); GAPDH (cytosolic marker); ERp72 (membrane marker); Lamin A+C (nuclear marker); H2A (chromatin marker); and vimentin (cytoskeletal marker). Molecular masses (kDa) are indicated on the left. Combinations tested were pCAG-empty plasmid plus PE243 (**A**); pCAG-empty plasmid plus Dak84 (**B**); pCAG-uORF2-FLAG plus PE243 (**C**); pCAG-African ORF-FLAG plus Dak84 (**D**); pCAG-mCherry plus PE243 (**E**) and pCAG-uORF1-mCherry plus PE243 (**F**). U251 cells were transfected with pCAG-mCherry (**G**) and pCAG-uORF1-mCherry (**H**) for 40 h p.t. and subjected to subcellular fractionation and analysis as described above. Experiments were performed in triplicate. Source data are provided as a Source Data file.

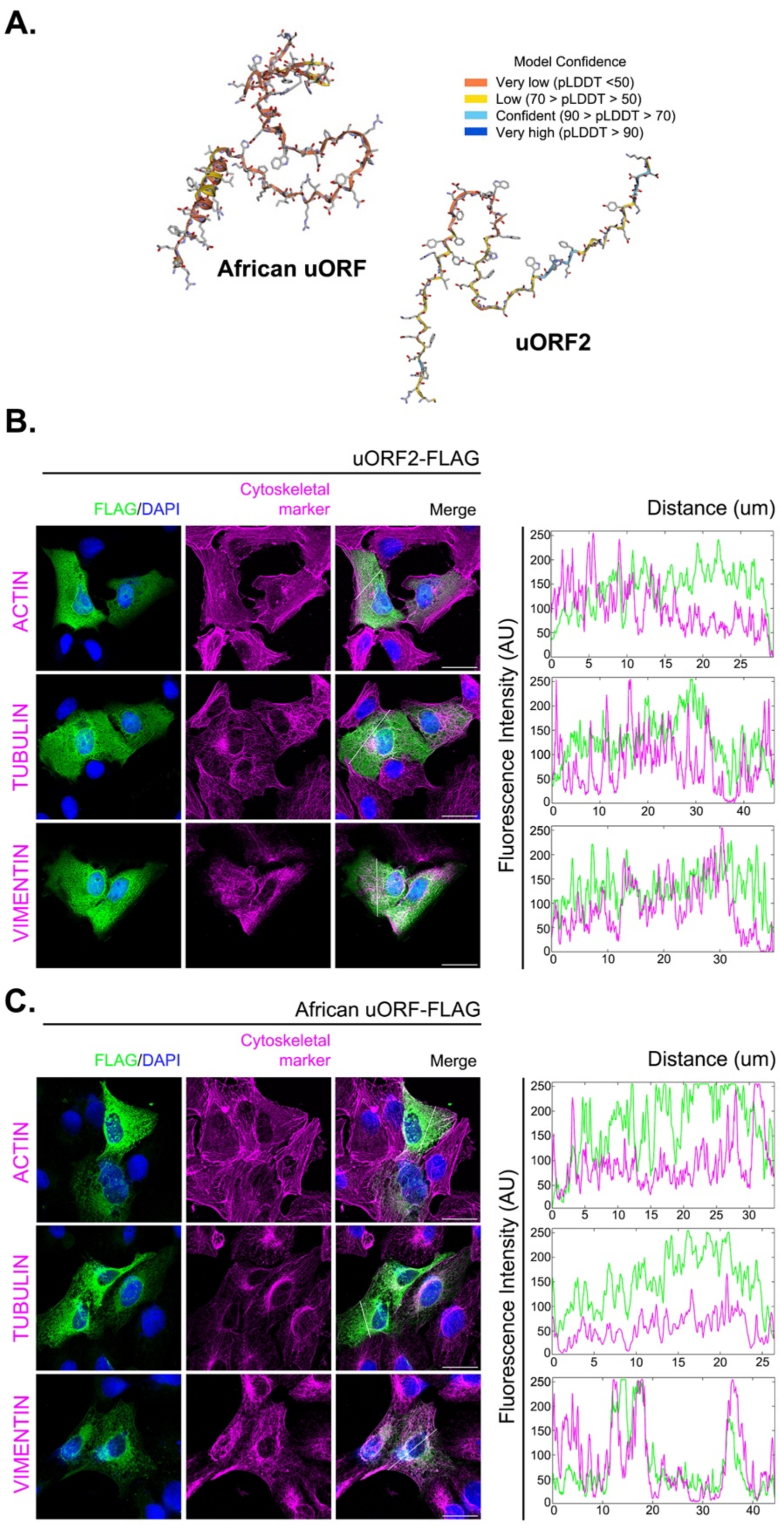

Supp. Figure 14. Asian/American uORF2 and African uORF structural prediction and subcellular localization. (**A**) AlphaFold2 predictions for Asian/American uORF2 and African uORF-encoded proteins. Representative confocal images of Vero cells transfected for 36 h

with pCAG-uORF2-FLAG (**B**) and pCAG-African uORF-FLAG (**C**) as described in **Fig 5B**. Experiments were performed in triplicate. Source data are provided as a Source Data file.

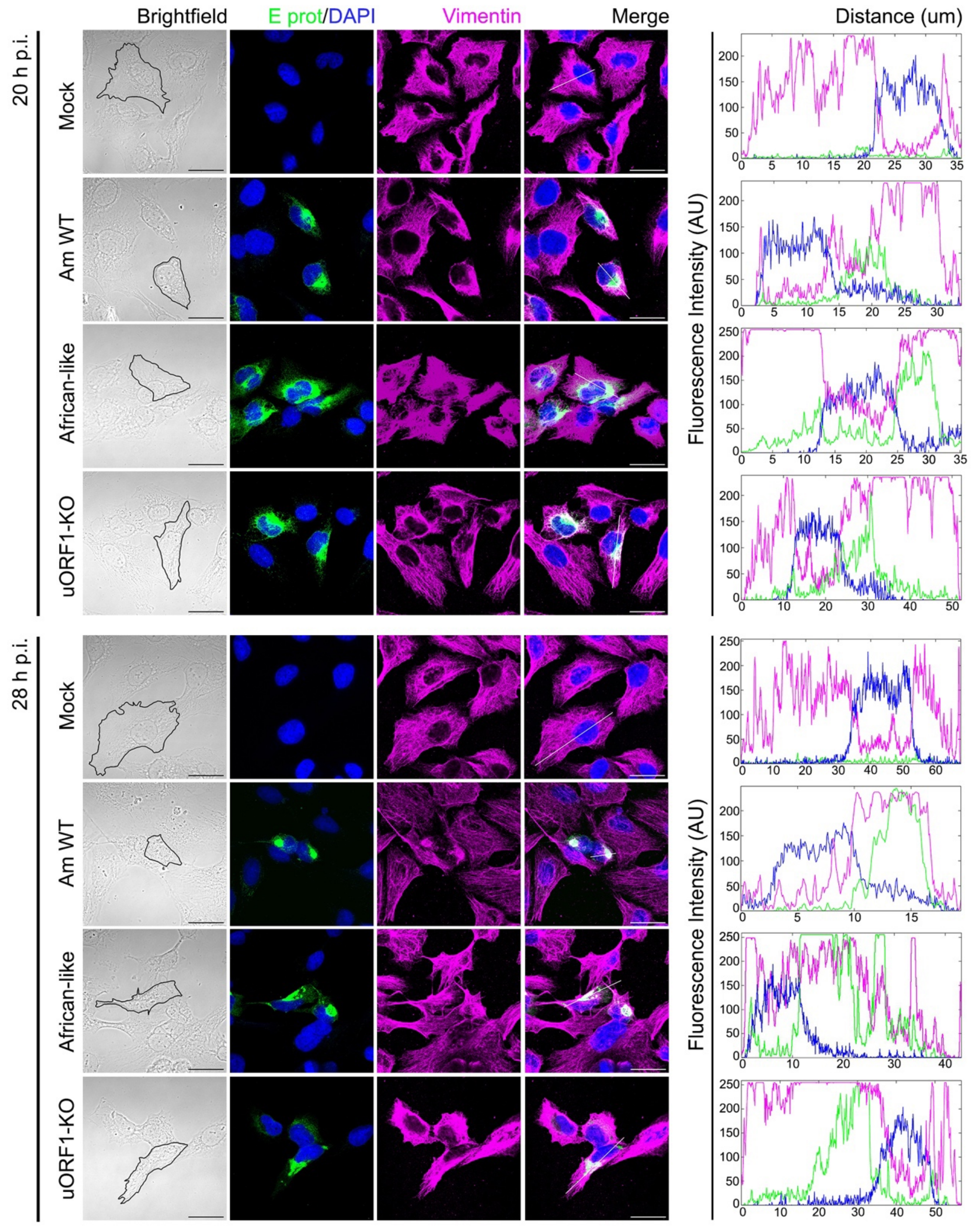

Supp. Figure 15. ZIKV uORF1-encoded protein helps in the formation of the cytoskeletal cage during infection. Representative brightfield and confocal images of U251 cells infected

with the American WT (Am WT), the African-like and the uORF1-KO mutant viruses (MOI:3) for 20h (upper panel) and 28 h (lower panel). Cells were stained and analysed as described in **Fig 6A**. Experiments were performed in triplicate. Source data are provided as a Source Data file.

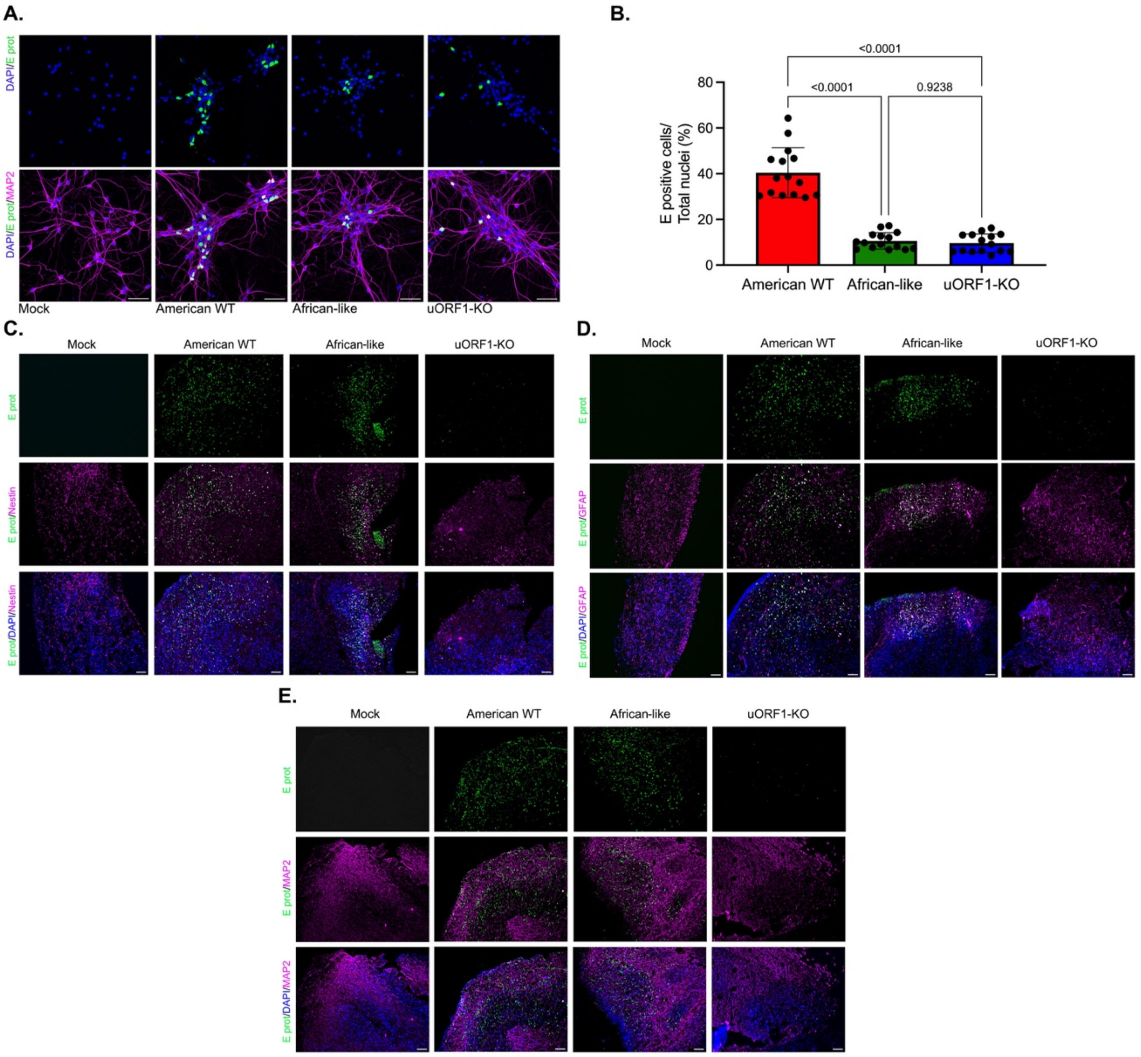

Supp. Figure 16. (**A**) Representative confocal images of i³Neurons infected with the American WT, the African-like and the uORF1-KO viruses (MOI:10) for 96 h. Cells were stained with antibodies against the viral E protein (green) and MAP2 (magenta), a marker for cortical neurons. Nuclei were counter-stained with DAPI (blue). Scale bars, 50 μm. (**B**) Percentage of E^+ cells in relation to total number of nuclei in i³Neurons infected with the American WT, the

African-like and the uORF1-KO viruses as described in **A**. 15 images per virus type were quantified. Error bars represent standard errors. Statistical analysis was one-way ANOVA with Gaussian distribution and did not assume equal variance for the two populations being compared. (**C**-**E**) Representative images (10X resolution) of ALI-COs infected with the American WT, the African-like and the uORF1-KO viruses (MOI:5) for 7 days showing immunoreactivity for the viral E protein (green) and different cellular markers (magenta), including nestin (**C**), GFAP (**D**) and MAP2 (**E**). Nuclei were counter-stained with DAPI (blue). Scale bars, 100 µm. Source data are provided as a Source Data file.

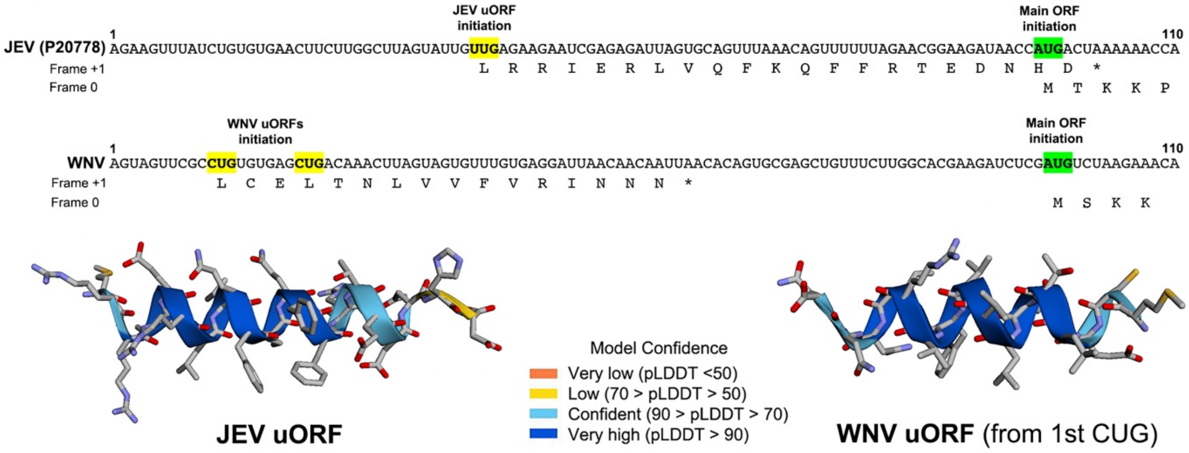

Supp. Figure 17. AlphaFold2 predictions for JEV uORF- and WNV uORF-encoded proteins. Ribosome profiling of JEV (P20778 strain) reveals translation of an uORF in the 5′ region using a non-canonical initiation codon (UUG in yellow)[5]. The JEV uORF-encoded peptide (primary sequence indicated in the +1 frame) is predicted to form an α-helix with high confidence as ZIKV uORF1. The 5′ region of WNV (accession number M12294.2) has two non-canonical initiation codons (in yellow) that could code for uORFs longer than 10 residues. The second CUG initiation codon aligns with the ZIKV uORF1 CUG initiation codon. The WNV uORF-encoded peptide initiating from the 1st CUG (primary sequence indicated in the

+1 frame) is also predicted with high confidence to form an α-helix. The AUG initiation codon for the main ORF translation is indicated in green.

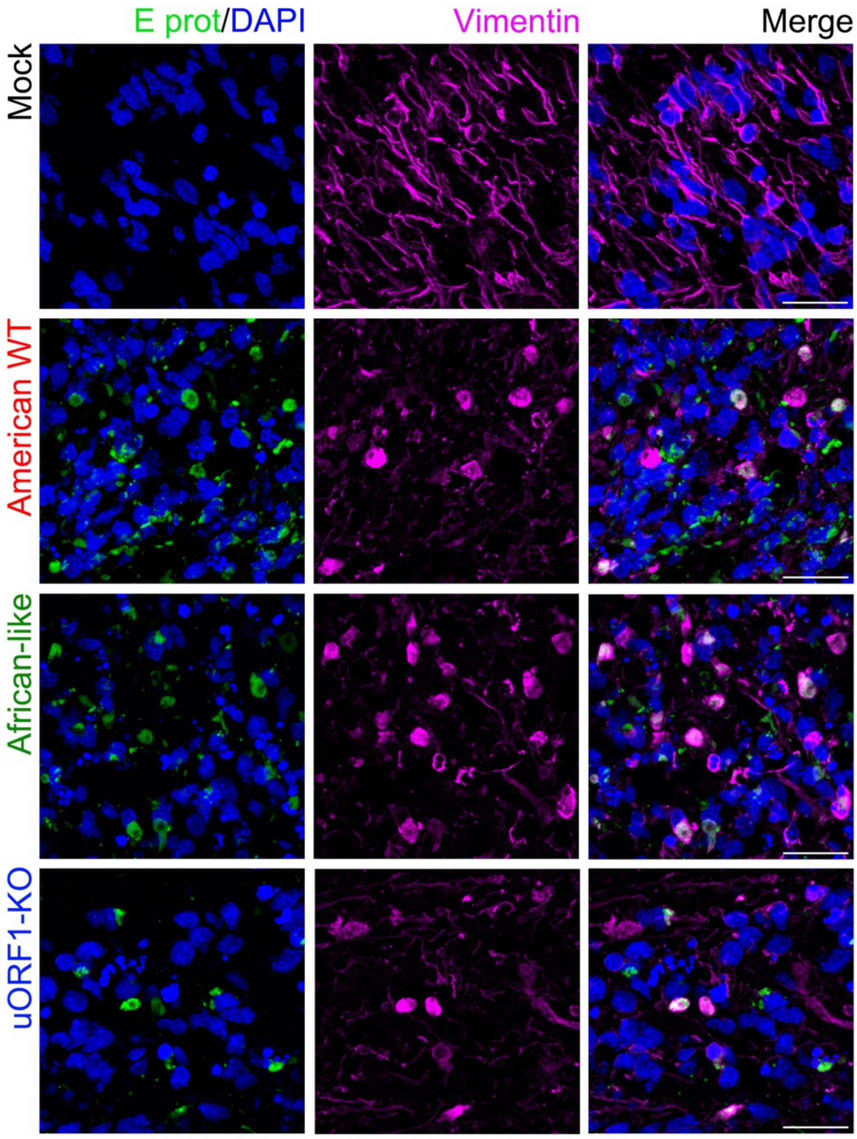

Supp. Figure 18. Collapse of vimentin in infected ALI-COs. Representative images of ALI-COs infected with the American WT, the African-like and the uORF1-KO viruses (MOI:5) for 7 days showing immunoreactivity for the viral E protein (green) and vimentin (magenta). Nuclei were counter-stained with DAPI (blue). Images represent the maximum projection of a Z-stack. Scale bars, 25 μm. Vimentin collapses in infected cells, this pattern is completely different to the filamentous one in mock-infected ALI-COs.

Supp Table 1. Zika virus (ZIKV) T7 luciferase reporters in Vero cells (raw luciferase data). Vero cells were reversed transfected with the corresponding T7 luciferase reporter and infected six hours later with the American strain PE243 (MOI:3) for 24 h.

Sample 1:

Only transfected cells

**FF-Luc**	**Rep1**	**Rep2**	**Rep3**	**Ren-Luc**	**Rep1**	**Rep2**	**Rep3**
**Mock**	20	20	40	**Mock**	50	100	50
**pREN**	80	50	90	**pREN**	258900	244100	265900
**ORF1**	569700	479500	429300	**ORF1**	307700	288300	283600
**ORF2**	1753000	2047000	1402000	**ORF2**	222900	281200	221400
**Main ORF**	36590000	34060000	32030000	**Main ORF**	313100	329300	316900

Transfected and infected cells

**FF-Luc**	**Rep1**	**Rep2**	**Rep3**	**Ren-Luc**	**Rep1**	**Rep2**	**Rep3**
**Mock**	20	40	30	**Mock**	80	60	70
**pREN**	90	50	50	**pREN**	289900	227200	317700
**ORF1**	430000	449600	338900	**ORF1**	166000	196400	151900
**ORF2**	2073000	1704000	1180000	**ORF2**	206000	212000	111900
**Main ORF**	27820000	33230000	24440000	**Main ORF**	191700	259500	181600

Sample 2:

Only transfected cells

**FF-Luc**	**Rep1**	**Rep2**	**Rep3**	**Ren-Luc**	**Rep1**	**Rep2**	**Rep3**
**Mock**	20	20	40	**Mock**	50	100	50
**pREN**	80	50	90	**pREN**	258900	244100	265900
**ORF1**	482400	463500	240400	**ORF1**	283000	288700	194300
**ORF2**	1538000	1535000	728900	**ORF2**	233900	290100	152300

**Main ORF**	24790000	27170000	16360000	**Main ORF**	299100	309600	215600

Transfected and infected cells

**FF-Luc**	**Rep1**	**Rep2**	**Rep3**	**Ren-Luc**	**Rep1**	**Rep2**	**Rep3**
**Mock**	20	40	30	**Mock**	80	60	70
**pREN**	90	50	50	**pREN**	289900	227200	317700
**ORF1**	383200	375200	428400	**ORF1**	191000	170700	213200
**ORF2**	1452000	1392000	1632000	**ORF2**	188200	151300	197700
**Main ORF**	21420000	20600000	25550000	**Main ORF**	184800	176400	222500

Sample 3:

Only transfected cells

**FF-Luc**	**Rep1**	**Rep2**	**Rep3**	**Ren-Luc**	**Rep1**	**Rep2**	**Rep3**
**Mock**	20	20	40	**Mock**	50	100	50
**pREN**	80	50	90	**pREN**	259000	244000	266000
**ORF1**	549700	335700	582200	**ORF1**	368500	250000	417000
**ORF2**	1887000	1136000	1855000	**ORF2**	369900	224800	366800
**Main ORF**	38470000	26420000	42680000	**Main ORF**	412300	301100	477400

Transfected and infected cells

**FF-Luc**	**Rep1**	**Rep2**	**Rep3**	**Ren-Luc**	**Rep1**	**Rep2**	**Rep3**
**Mock**	20	40	30	**Mock**	80	60	70
**pREN**	90	50	50	**pREN**	290000	227000	318000
**ORF1**	568400	589700	341700	**ORF1**	263800	265100	191900
**ORF2**	1640000	1528000	1278000	**ORF2**	257400	198100	178300
**Main ORF**	28210000	17940000	17440000	**Main ORF**	218700	154800	171400

Supp Table 2. Percentage of ZIKV E-positive cells in relation to total number of nuclei.

33 images per virus type at 20X resolution (approx. 400-500 nuclei/image) were quantified for E-positive staining. SDEV represent standard deviation.

Image Number	American WT (% infected cells)	African-like (% infected cells)	uORF1-KO (% infected cells)
**1**	45.17045455	42.30769231	7.478632479
**2**	53.57142857	22.23650386	7.142857143
**3**	57.58196721	48.00796813	10.20408163
**4**	33.96226415	24.37574316	10.29810298
**5**	63.82978723	26.24277457	7.63546798
**6**	57.71144279	37.5984252	4.295942721
**7**	53.77777778	45.56521739	6.497175141
**8**	48.77300613	41.84397163	7.203389831
**9**	48.01223242	34.24657534	2.30125523
**10**	32.36151603	26.05752961	7.2319202
**11**	53.38345865	25.79666161	10.92592593
**12**	39.83228512	35.66433566	Bad scanning
**13**	48.0127186	35.34482759	10.17699115
**14**	38.60103627	41.39534884	6.161137441
**15**	37.55274262	32.70348837	8.613445378
**16**	32.46492986	28.85714286	10.20408163
**17**	36.73110721	36.62650602	18.02469136
**18**	40.06024096	26.30853994	17.35751295
**19**	34.00503778	26.8683274	9.282700422
**20**	36.38613861	23.94957983	19.18103448
**21**	21.25237192	30.41749503	14.89361702
**22**	45.71428571	27.30318258	16.04197901
**23**	25.18853695	26.92307692	12.72264631
**24**	55.46558704	28.63070539	5.882352941
**25**	37.42138365	25.40106952	11.37640449
**26**	51.36054422	32.42105263	17.09401709
**27**	46.32911392	25.08591065	8.603351955
**28**	52.38095238	28.55329949	16.76792224
**29**	62.25490196	37.98283262	15.90909091
**30**	67.47211896	38.89845095	9.172482552
**31**	79.83651226	40.83175803	14.30921053
**32**	71.38047138	44.22310757	8.421052632
**33**	52.60223048	31.54362416	11.5942029
**Average**	**47.28607828**	**32.73371894**	**10.71889615**
**STED**	**13.2234658**	**7.217031425**	**4.355901449**

Supp Table 3. Percentage of ZIKV E-positive cells that are positive for different cellular markers infected with different ZIKV mutant viruses:

SDEV indicates standard deviation.

a. % Cells that are positive for E and nestin

Image number	American WT (%)	African-like (%)	uORF1-KO (%)
**1**	38.99371069	35.35353535	40.000000000
**2**	33.84615385	41.04046243	42.424242424
**3**	36.29893238	43.98340249	46.000000000
**4**	36.11111111	44.3902439	44.736842105
**5**	41.11111111	35.24229075	45.161290323
**6**	34.34343434	47.11111111	26.811594203
**7**	38.05774278	31.63841808	17.142857143
**8**	34.15977961	19.91150442	45.652173913
**9**	26.62116041	34.25925926	25.287356322
**10**	43.39622642	43.24324324	36.111111111
**11**	28.97526502	39.89361702	31.250000000
**Average**	**35.62860252**	**37.82428073**	**36.41613341**
**SDEV**	**4.905994782**	**7.719430605**	**9.930490336**

b. % Cells that are positive for E and MAP2

Image number	American WT (%)	African-like (%)	uORF1-KO (%)
**1**	1.293103448	2.094240838	0.000000000
**2**	2.479338843	1.145038168	4.347826087
**3**	1.257861635	0.847457627	2.941176471
**4**	2.547770701	1.142857143	0.000000000
**5**	3.603603604	1.948051948	0.000000000
**6**	2.604166667	4.90797546	6.542056075
**7**	3.592814371	4.761904762	8.000000000
**8**	6.569343066	5.797101449	6.896551724
**9**	6.722689076	6.315789474	9.876543210
**10**	7.284768212	1.948051948	10.000000000
**11**	7.650273224	4.109589041	6.493506494
**Average**	**4.145975713**	**3.183459805**	**5.008878187**
**SDEV**	**2.43790138**	**2.024370548**	**3.816402741**

c. % Cells that are positive for E and GFAP

Image number	American WT (%)	African-like (%)	uORF1-KO (%)
1	27.69953052	31.76470588	28.81355932
2	22.63157895	28.62745098	Bad scanning
3	32.45033113	29.26829268	36.95652174
4	35.5704698	32.02247191	42.30769231
5	28.08988764	32.88888889	31.70731707
6	20.37037037	36.63366337	25.00000000
7	23.44497608	32.89473684	32.87671233
8	30.82706767	32.46073298	34.32835821
9	21.48148148	25.82781457	34.09090909
10	31.97278912	35.0877193	26.96629213
11	32.14285714	30.71895425	30.15873016
**Average**	**27.88012181**	**31.65413015**	**32.32060924**
**SDEV**	**5.179448745**	**3.002016735**	**5.036721375**

Supp Table 4. Total number of analysed and ZIKV positive *Ae. aegypti* mosquitoes per experiment and time points assessing infection, dissemination and transmission prevalence of different ZIKV mutant viruses.

**Infection prevalence**		Day 7			Day 10			Day 14			Day 17			Day 21		
Experiment	Virus	total	positive	ratio	total	positive	ratio	total	positive	ratio	total	positive	ratio	total	positive	ratio
	WT	11	10	0.91	-	-	-	18	17	0.94	-	-	-	5	5	1
#1	Africa	15	10	0.67	-	-	-	9	8	0.89	-	-	-	2	2	1
	uORF1	22	19	0.86	-	-	-	10	9	0.90	-	-	-	7	7	1
	WT	8	8	1.00	11	10	0.91	13	12	0.92	9	7	0.78	-	-	-
#2	Africa	9	8	0.89	8	7	0.88	10	9	0.90	12	10	0.83	-	-	-
	uORF1	10	10	1.00	12	11	0.92	11	9	0.82	15	14	0.93	-	-	-
	WT	12	10	0.83	19	19	1.00	18	15	0.83	10	10	1.00	-	-	-
#3	Africa	25	23	0.92	24	23	0.96	28	22	0.79	14	11	0.79	-	-	-
	uORF1	25	21	0.84	26	26	1.00	23	22	0.96	10	10	1.00	-	-	-
	WT	31	28	0.90	30	29	0.97	49	44	0.90	19	17	0.89	5	5	1
Total	Africa	49	41	0.84	32	30	0.94	47	39	0.83	26	21	0.81	2	2	1
	uORF1	57	50	0.88	38	37	0.97	44	40	0.91	25	24	0.96	7	7	1

**Dissemination prevalence**		Day 7			Day 10			Day 14			Day 17			Day 21		
Experiment	Virus	total	positive	ratio	total	positive	ratio	total	positive	ratio	total	positive	ratio	total	positive	ratio
	WT	10	7	0.70	-	-	-	17	15	0.88	-	-	-	5	5	1
#1	Africa	10	9	0.90	-	-	-	8	7	0.88	-	-	-	2	2	1
	uORF1	19	14	0.74	-	-	-	9	8	0.89	-	-	-	7	7	1
	WT	8	6	0.75	10	10	1.00	12	12	1.00	7	7	1.00	-	-	-
#2	Africa	8	8	1.00	7	5	0.71	9	8	0.89	10	10	1.00	-	-	-
	uORF1	10	8	0.80	11	10	0.91	9	9	1.00	14	14	1.00	-	-	-
	WT	10	9	0.90	19	17	0.89	15	14	0.93	10	10	1.00	-	-	-
#3	Africa	23	12	0.52	23	16	0.70	22	21	0.95	11	11	1.00	-	-	-
	uORF1	21	17	0.81	26	19	0.73	22	21	0.95	10	10	1.00	-	-	-
	WT	28	22	0.79	29	27	0.93	44	41	0.93	17	17	1.00	5	5	1
Total	Africa	41	29	0.71	30	21	0.70	39	36	0.92	21	21	1.00	2	2	1
	uORF1	50	39	0.78	37	29	0.78	40	38	0.95	24	24	1.00	7	7	1

**Transmission prevalence**		Day 7			Day 10			Day 14			Day 17			Day 21		
Experiment	Virus	total	positive	ratio	total	positive	ratio	total	positive	ratio	total	positive	ratio	total	positive	ratio
	WT	7	0	0.00	-	-	-	15	4	0.27	-	-	-	5	2	0.40
#1	Africa	9	0	0.00	-	-	-	7	0	0.00	-	-	-	2	1	0.50
	uORF1	14	3	0.21	-	-	-	8	0	0.00	-	-	-	7	1	0.14
	WT	6	0	0.00	10	2	0.20	12	3	0.25	7	1	0.14	-	-	-
#2	Africa	8	0	0.00	5	0	0.00	8	3	0.38	10	2	0.20	-	-	-
	uORF1	8	0	0.00	10	1	0.10	9	1	0.11	14	2	0.14	-	-	-
	WT	9	0	0.00	17	3	0.18	14	0	0.00	10	2	0.20	-	-	-
#3	Africa	12	0	0.00	16	0	0.00	21	1	0.05	11	0	0.00	-	-	-
	uORF1	17	0	0.00	19	0	0.00	21	0	0.00	10	1	0.10	-	-	-
	WT	22	0	0.00	27	5	0.19	41	7	0.17	17	3	0.18	5	2	0.40
Total	Africa	29	0	0.00	21	0	0.00	36	4	0.11	21	2	0.10	2	1	0.50
	uORF1	39	3	0.08	29	1	0.04	38	1	0.03	24	3	0.13	7	1	0.14

Supp Table 5. Test statistics of ZIKV infection, dissemination and transmission in *Ae. aegypti*. The table shows the logistic regression analysis results using likelihood ratio tests to assess the statistical significance of effects. There is no adjustment for multiple comparisons because the model is only run once for each phenotype. The logistic regression test compares the fit of a full model with all predictors to a reduced model (often the null model with only the intercept). The test statistic follows a chi-square distribution with degrees of freedom equal to the difference in the number of parameters between the full and reduced models. A significant result indicates that the full model provides a significantly better fit to the data than the reduced model. Interactions terms were removed from the final model because their effect was non-significant (P>0.05). Blood meal titre was $\log_{10}$-transformed.

		Infection		Dissemination		Transmission	
Variable	df	LR Chi^2	P value	LR Chi^2	P value	LR Chi^2	P value
Blood meal titre	1	0.3471	0.5558	0.9425	0.3316	0.1938	0.6598
Experiment	2	1.4035	0.4957	4.4994	0.1054	8.8575	0.0119
Virus strain	2	4.8015	0.0906	4.3105	0.1159	4.9545	0.0840
Time	1	0.3726	0.5416	28.529	<0.0001	6.0300	0.0141

df: degrees of freedom; LR=likelihood ratio.

Supp Table 6. Percentage (calculated from sequencing data) **of ZIKV mutant viruses in competition assays in U251 and Vero cells.**

U251 cells

American WT (50%): African-like (50%)

**American WT**	**Rep1**	**Rep2**	**Rep3**	**Rep4**	**Rep5**	**Rep6**	**Rep7**	**Rep8**	**Average**
**P0 (Input)**	50								50
**P2**	11.67	25.40	17.15	20.36	8.07	10.56	10.71	10.17	14.26
**P4**	5.78	22.24	4.61	7.27	4.93	5.61	4.95	5.07	7.56
**P6**	6.33	18.57	3.43	4.18	8.13	21.31	8.45	11.20	10.20

**African-like**	**Rep1**	**Rep2**	**Rep3**	**Rep4**	**Rep5**	**Rep6**	**Rep7**	**Rep8**	**Average**
**P0 (Input)**	50								50
**P2**	88.37	74.60	82.85	79.64	91.93	89.44	89.29	89.83	85.74
**P4**	94.22	77.76	95.39	92.73	95.07	94.39	95.05	94.93	92.44
**P6**	93.67	81.43	96.57	95.82	91.87	78.69	91.55	88.80	89.80

American WT (90%): African-like (10%)

**American WT**	**Rep1**	**Rep2**	**Rep3**	**Rep4**	**Rep5**	**Rep6**	**Rep7**	**Rep8**	**Average**
**P0 (Input)**	90								90
**P2**	48.74	63.57	56.37	58.77	43.35	32.90	36.13	43.42	47.91
**P4**	42.83	28.27	19.70	17.65	19.10	8.59	11.85	14.94	20.37
**P6**	14.30	19.20	15.15	10.06	15.07	8.21	11.95	11.29	13.16

**African-like**	**Rep1**	**Rep2**	**Rep3**	**Rep4**	**Rep5**	**Rep6**	**Rep7**	**Rep8**	**Average**
**P0 (Input)**	10								10
**P2**	51.26	36.43	43.63	41.23	56.65	67.10	63.87	56.58	52.09
**P4**	57.17	71.73	80.30	82.35	80.90	91.41	88.15	85.06	79.63
**P6**	85.70	80.80	84.85	89.94	84.93	91.79	88.05	88.71	86.84

American WT (50%): uORF1-KO (50%)

**American WT**	**Rep1**	**Rep2**	**Rep3**	**Rep4**	**Rep5**	**Rep6**	**Rep7**	**Rep8**	**Average**
**P0 (Input)**	50								50
**P2**	18.93	59.54	39.68	46.20	30.61	36.89	34.65	35.38	37.74
**P4**	29.82	46.12	N/A	23.71	22.02	19.81	16.72	30.32	26.93
**P6**	32.80	39.86	16.71	31.44	12.11	7.15	15.18	37.61	24.11

uORF1-KO	Rep1	Rep2	Rep3	Rep4	Rep5	Rep6	Rep7	Rep8	Average
**P0 (Input)**	50								50
**P2**	81.07	40.46	60.32	53.80	69.39	63.11	65.35	64.62	62.26
**P4**	70.18	53.88	N/A	76.29	77.98	80.19	83.28	69.68	73.07
**P6**	67.20	60.14	83.29	68.56	87.89	92.85	84.82	62.39	75.89

American WT (90%): uORF1-KO (10%)

American WT	Rep1	Rep2	Rep3	Rep4	Rep5	Rep6	Rep7	Rep8	Average
**P0 (Input)**	90								90
**P2**	63.07	66.60	65.16	68.32	68.13	68.07	74.10	67.44	67.61
**P4**	2.33	5.44	5.60	5.28	1.62	9.20	6.44	2.06	4.75
**P6**	1.19	0	1.08	0	5.28	0.20	0.52	0	1.03

uORF1-KO	Rep1	Rep2	Rep3	Rep4	Rep5	Rep6	Rep7	Rep8	Average
**P0 (Input)**	10								10
**P2**	36.93	33.40	34.84	31.68	31.87	31.93	25.90	32.56	32.39
**P4**	97.67	94.56	94.40	94.72	98.38	90.80	93.56	97.94	95.25
**P6**	98.81	100	98.92	100	94.72	99.80	99.48	100	98.97

American WT (50%): uORF2-PTC1 (50%)

American WT	Rep1	Rep2	Rep3	Rep4	Rep5	Rep6	Rep7	Rep8	Average
**P0 (Input)**	50								50
**P2**	54.02	23.92	20.92	20.95	19.97	17.27	25.83	19.30	25.27
**P4**	37.93	31.40	32.95	23.97	20.17	17.26	19.70	22.16	25.69
**P6**	75.88	58.24	70.97	34.24	44.91	23.11	32.38	35.20	46.87

uORF2-PTC1	Rep1	Rep2	Rep3	Rep4	Rep5	Rep6	Rep7	Rep8	Average
**P0 (Input)**	50								50
**P2**	45.98	76.08	70.08	79.05	80.03	82.73	74.17	80.70	74.73
**P4**	62.07	68.60	67.05	76.03	79.83	82.74	80.30	77.84	74.31
**P6**	24.12	41.76	29.03	65.76	55.09	76.89	67.62	64.80	53.13

American WT (10%): uORF2-PTC1 (90%)

American WT	Rep1	Rep2	Rep3	Rep4	Rep5	Rep6	Rep7	Rep8	Average
**P0 (Input)**	10								10
**P2**	16.67	44.52	21.72	15.48	7.61	5.65	5.28	4.43	15.17
**P4**	8.54	5.26	3.61	5.33	3.38	5.74	5.03	5.19	5.26
**P6**	2.72	5.28	3.99	4.07	5.96	9.56	5.67	10.40	5.96

uORF2-PTC1	Rep1	Rep2	Rep3	Rep4	Rep5	Rep6	Rep7	Rep8	Average
**P0 (Input)**	90								90
**P2**	83.33	55.48	78.28	84.52	92.39	94.35	94.72	95.57	84.83
**P4**	91.46	94.74	96.39	94.67	96.62	94.26	94.97	94.81	94.74
**P6**	97.28	94.72	96.01	95.93	94.04	90.44	94.33	89.60	94.04

Vero cells

American WT (50%): African-like (50%)

American WT	Rep1	Rep2	Rep3	Rep4	Rep5	Rep6	Rep7	Rep8	Average
**P0 (Input)**	50								50
**P2**	26.23	35.81	39.51	32.56	39.83	27.50	28.45	27.95	32.23
**P4**	21.88	18.63	25.39	27.00	27.71	24.73	25.14	26.97	24.68
**P6**	23.79	11.50	11.39	10.64	17.57	15.72	17.55	15.67	15.48

African-like	Rep1	Rep2	Rep3	Rep4	Rep5	Rep6	Rep7	Rep8	Average
**P0 (Input)**	50								50
**P2**	73.77	64.19	60.49	67.44	60.17	72.50	71.55	72.05	67.77
**P4**	78.12	81.37	74.61	73.00	72.29	75.27	74.86	73.03	75.32
**P6**	76.21	88.50	88.61	89.36	82.43	84.28	82.45	84.33	84.52

American WT (50%): uORF1-KO (50%)

American WT	Rep1	Rep2	Rep3	Rep4	Rep5	Rep6	Rep7	Rep8	Average
**P0 (Input)**	50								50
**P2**	42.55	42.83	45.87	46.61	48.91	49.44	48	48.76	46.62
**P4**	51.79	27.61	35.79	48.49	46.98	53.04	41.36	45.43	43.81
**P6**	33.96	19.54	23.68	31.62	45.89	50.14	39.36	46.37	36.32

uORF1-KO	Rep1	Rep2	Rep3	Rep4	Rep5	Rep6	Rep7	Rep8	Average
**P0 (Input)**	50								50
**P2**	57.45	57.17	54.13	53.39	51.09	50.56	52	51.24	53.38
**P4**	48.21	72.39	64.21	51.51	53.02	46.96	58.64	54.57	56.19
**P6**	66.04	80.46	76.32	68.38	54.11	49.86	60.64	53.63	63.68

American WT (50%): uORF2-PTC1 (50%)

American WT	Rep1	Rep2	Rep3	Rep4	Rep5	Rep6	Rep7	Rep8	Average
**P0 (Input)**	50								50
**P2**	67.05	59.23	62.41	71.24	58.32	59.87	61.34	62.21	62.71
**P4**	80.29	73.10	73.39	69.83	59.43	67.43	68.94	73.14	70.69
**P6**	N/A	74.02	70	75.99	70.51	72.40	76.18	65.19	72.04

uORF2-PTC1	Rep1	Rep2	Rep3	Rep4	Rep5	Rep6	Rep7	Rep8	Average
**P0 (Input)**	50								50
**P2**	32.95	40.77	37.59	28.76	41.68	40.13	38.66	37.79	37.29
**P4**	19.71	26.90	26.61	30.17	40.57	32.57	31.06	26.86	29.31
**P6**	N/A	25.98	30	24.01	29.49	27.60	23.82	34.81	27.96

Supplementary Table 7. List of oligonucleotides; *fwd* (forward) and *rev* (reverse) primers.

Name	Sequence (5′-3′)
uORF1 KO, fwd	TGTGTGAATCAGACTACGACAGTTCGAGTTT
uORF1 KO, rev	AAACTCGAACTGTCGTAGTCTGATTCACACA
African ORF like, fwd	CAGTATCAACAGGTTTAATTTGGATTTGGAAACGAGA
African ORF like, rev	TCTCGTTTCCAAATCCAAATTAAACCTGTTGATACTG
uORF2 KO, fwd	AACAGGTTTTATTTTAGATTTGGAAACGAGA
uORF2 KO, rev	TCTCGTTTCCAAATCTAAAATAAAACCTGTT
uORF2-AUG, fwd	AACAGGTTTTATTATGGATTTGGAAACGAGA
uORF2-AUG, rev	TCTCGTTTCCAAATCCATAATAAAACCTGTT
uORF2-PTC1, fwd	TTGGATTTGGAAACGTGAGTTTCTGGTCATG
uORF2-PTC1, rev	CATGACCAGAAACTCACGTTTCCAAATCCAA
uORF1 in frame pSGD, fwd	GCGCCTCGAGATAAGTTGTTGATCTGTGTGAATCAGAC
uORF1 in frame pSGD, rev	GCGCAGATCTATTTCTTTTTTGGGGTTTTCCATGACCAG
uORF2 in frame pSGD, fwd	GCGCCTCGAGATAAGTTGTTGATCTGTGTGAATCAGAC
uORF2 in frame pSGD, rev	GCGCAGATCTAAGCCCCCAAAGGGGCTCACACGGG
Main ORF in frame pSGD, fwd	GCGCCTCGAGATAAGTTGTTGATCTGTGTGAATCAGAC
Main ORF in frame pSGD, rev	GCGCAGATCTGCCCCCAAAGGGGCTCACACGGGCTACTCCG
T7 upstream of ZIKV uORFs, fwd	GACTCACTATAGGGAGTTGTTGATCTGTGTGAATC
T7 upstream of ZIKV uORFs, rev	GATTCACACAGATCAACAACTCCCTATAGTGAGTC
uORF2-PTC34, fwd	CGGAGTAGCCCGTGTTAGCCCCTTTGGGGGC
uORF2-PTC34, rev	GCCCCCAAAGGGGCTAACACGGGCTACTCCG
uORF2-FS1, fwd	CGAGAGTTTCTGGTCTTAGAAAACCCAAAAAAGA
uORF2-FS1, rev	TCTTTTTTGGGTTTTCTAAGACCAGAAACTCTCG
uORF2-FS2, fwd	GTTTCTGGTCATGAAGAACCCAAAAAAGAAA
uORF2-FS2, rev	TTTCTTTTTTGGGTTCTTCATGACCAGAAAC
uORF2-FS3, fwd	GGTCATGAAAAACCCGAAGAAGAAATCCGGAGGA
uORF2-FS3, rev	TCCTCCGGATTTCTTCTTCGGGTTTTTCATGACC
uORF2-alternative initiation Mut1, fwd	AAACCCAAA AAAGAAGTCCGGAGGATTCCGG
uORF2-alternative initiation Mut1, rev	CCGGAATCCTCCGGACTTCTTTTTTGGGTTT
uORF2-alternative initiation Mut2, fwd	A AAAGAAGTCCGGAGAATTCCGGATTGTCAA
uORF2-alternative initiation Mut2, rev	TTGACAATCCGGAATTCTCCGGACTTCTTTT
uORF2-alternative initiation Mut3, fwd	AAAGAAGTCCGGAGAGTTCCGGATTGTCAA T
uORF2-alternative initiation Mut3, rev	ATTGACAATCCGGAACTCTCCGGACTTCTTT
uORF2-alternative initiation Mut4, fwd	TCAATATGCTAAAACACGGAGTAGCCCGTGT
uORF2-alternative initiation Mut4, rev	ACACGGGCTACTCCGTGTTTTAGCATATTGA
uORF1-TAP in pCAG, fwd	GCGCTTAATTAAACCATGCGACAGTTCGAGTTTGAAGC
uORF1-TAP in pCAG, rev	GCGCCTTAAGTTACTTGTCATCGTCATCCTTG
uORF1-mCherry in pCAG (PCR1), fwd	GCGCTTAATTAAACCATGCGACAGTTCGAGTTTGAAGCG
uORF1-mCherry in pCAG (PCR1), rev	GTTATCCTCCTCGCCCTTGCTCACGGCTGATGACCAGAAACTCTC GTTTCCAAA
uORF1-mCherry in pCAG (PCR2), fwd	TTTGGAAACGAGAGTTTCTGGTCATCAGCCGTGAGCAAGGGCG AGGAGGATAAC
uORF1-mCherry in pCAG (PCR2), rev	GCGCCTTAAGTTACTTGTACAGCTCGTCCATGCCGCC
uORF2-FLAG in pCAG, fwd	GCGCTTAATTAAACCATGGATTTGGAAACGAGAGTTTC
uORF2-FLAG in pCAG, rev	GCGCCTTAAGCTACTTGTCGTCATCGTCTTTGTAGTCTTGATGAG ACCCAGTGATGGC
African ORF-FLAG in pCAG, fwd	GCGCTTAATTAAACCATGCGACAGTTCGAGTTTGAAGC
African ORF-FLAG in pCAG, rev	GCGCCTTAAGCTACTTGTCGTCATCGTCTTTGTAGTCTTGATGAG ACCCAGTGATGGC
SHAPE analysis, fwd	GGCTACTCCGCGTTTTAGCATATTG
SHAPE analysis, rev	TCTTCAAGCCCCCAAAGGGGCTCAC
To amplify ZIKV 5′ UTR, fwd	GTTGTTGATCTGTGTGAATCAG
To amplify ZIKV 5′ UTR, rev	TATTGATGAGACCCAGTGATGGC
To sequence ZIKV 5′ UTR, rev	GACCCAGCAGAAGTCCGGCTGGC
uORF1-TAP-1X Pro, fwd	GCGAAAGCTAGCAACAGTCCCAACAGGTTTATTTTGGA TTT
uORF1-TAP-1X Pro, rev	AAATCCAAAATAAAACCTGTTGGGACTGTTGCTAGCTTTC GC

Supplementary Table 8. List of antibodies used.

- **Immunoblotting**

Antibody	Host	Type	Source	Dilution
		**Primary Antibodies**		
Anti-GAPDH	Mouse	IgM	Sigma-Aldrich, G8795	1:20,000
Anti-FLAG	Mouse	IgG	Sigma-Aldrich, SAB4301135	1:2,000
Anti-E protein	Rabbit	IgG	GeneTex, GTX133314	1:1,000
Anti-Lamin A+C	Rabbit	IgG	Abcam, ab108922	1:1,000
Anti-H2A	Rabbit	IgG	Abcam, ab1777308	1:1,000
Anti-Vimentin	Mouse	IgG1	Abcam, ab8069	1:1,000
Anti-ERp72	Rabbit	IgG	Cell Signaling, 5033	1:1,000
Anti-eIF2α	Rabbit	IgG	Cell Signaling, 9722	1:1,000
Anti-p-eIF2α	Rabbit	IgG	Cell Signaling, 9721	1:1,000
Anti-mCherry	Rabbit	IgG	Abcam, ab167453	1:1,000
		**Secondary Antibodies**		
Anti-Rabbit	Goat	800	Licor, IRDye 926-32211	1:1,000
Anti-Mouse IgM	Donkey	680	Licor, IRDye 926-68180	1:1,000
Anti-Mouse	Goat	800	Licor, IRDye 926-32210	1:1,000

- **Immunofluorescence**

Antibody	Host	Type	Source, Catalogue #	Dilution
		**Primary Antibodies**		
Anti-E protein	Rabbit	IgG	GeneTex, GTX133314	1:200
Anti-flavivirus Ag group	Mouse	IgG	Merck, D1-4G2-4-15	1:200
Anti-flavivirus Ag group	Rabbit	IgG	Absolute antibody, Ab00230-23.0	1:200
Anti-FLAG	Mouse	IgG	Sigma-Aldrich, SAB4301135	1:500
Anti-Vimentin	Mouse	IgG1	Abcam, ab8069	1:500
Anti-Actin	Mouse	IgG	Proteintech, 66009	1:500
Anti-Tubulin	Rat	Hybridoma	Kind gift from Dr Colin Crump	1:10
Anti-MAP2	Goat	IgG	antibodies.com, A104327	1:1,000
Anti-Nestin	Mouse	IgG1	Abcam, ab22035	1:500

Anti-GFAP	Rabbit	IgG	antibodies.com, A85419	1:1,000
Anti-FLAG	Rabbit	IgG	Sigma-Aldrich, 740001	1:100
		**Secondary Antibodies**		
Anti-Rabbit	Goat	488	Invitrogen Alexa Fluor, A11008	1:1,000
Anti-Rabbit	Donkey	488	Abcam, ab150073	1:1,000
Anti-Mouse	Goat	488	Invitrogen Alexa Fluor, A11001	1:1,000
Anti- Rabbit	Donkey	594	Invitrogen Alexa Fluor, A21207	1:1,000
Anti-Mouse	Donkey	594	Invitrogen Alexa Fluor, A21203	1:1,000
Anti-Rat	Goat	568	Invitrogen Alexa Fluor, A11077	1:1,000

Supp Table 9. Read counts for RNA-Seq and Ribo-Seq samples

Sample	Total Number	rRNA		vRNA		mRNA		ncRNA		gDNA		Contaminants	
		Pos.	*Neg.*	*Pos.*	*Neg.*	*Pos.*	*Neg.*	*Pos.*	*Neg.*	*Pos.*	*Neg.*	*Pos.*	*Neg.*
**PE243_Ribo_Vero_CHX_REP1**	31997688	20313714	24586	81496	191	9047129	42705	140885	552	327237	104116	35	78
**PE243_RNA_Vero_CHX_REP1**	22731710	803760	106898	81423	18377	3219832	3190284	8942148	20052	1980011	1500308	172	620
**PE243_Ribo_Vero_CHX_REP2**	37222210	29458213	4786	55842	44	5643412	24525	96617	365	300795	71980	17	37
**PE243_RNA_Vero_CHX_REP2**	18721434	784934	79268	80377	18648	2945818	2637022	7360285	13298	1627753	1213924	91	346
**PE243_Ribo_Vero_REP1**	135911569	127112183	90723	144171	53	2163829	64139	340677	1224	564184	91069	335	884
**PE243_RNA_Vero_REP1**	32886637	1031137	110961	538022	5380	4408320	5276292	12872242	21509	3175146	2082474	971	1907
**PE243_Ribo_Vero_REP2**	31073211	28747554	16590	39155	15	842719	18946	98967	496	183022	31701	263<br>1295	706<br>1110
**PE243_RNA_Vero_REP2**	36901188	1072272	120592	548739	4422	4942467	5885041	15008616	34344	3612077	2422266	725<br>1729	1388<br>1363
**Dak84_Ribo_Vero_REP1**	32836601	12763206	213508	20977	28	797821	353061	1744176	5988	976810	307178	725<br>7116952	1718<br>6245759
**Dak84_RNA_Vero_REP1**	33319794	712578	43059	25191	716	1554526	5821179	12176367	10959	2981969	1963087	442<br>2328134	875<br>2967305
**Dak84_Ribo_Vero_REP2**	39316952	9227360	922056	10344	47	554277	359347	2166158	9362	2676928	519297	2926<br>10278950	7180<br>6795836
**Dak84_RNA_Vero_REP2**	32653637	521889	54192	81987	750	2018383	374350	9826321	17317	2194009	1566586	934<br>4243431	917<br>5563149
**PE243_Ribo_U251**	51487828	16715591	1710511	498979	2531	22551957	202743	1729410	110261	450661	769386	9049	21956
**PE243_RNA_U251**	51593828	484481	117611	783443	32425	10198994	5250036	11928481	591128	5898754	4651609	3714	9681
**Dak84_Ribo_U251**	55661825	25171380	1013624	503463	49078	4831087	788056	1625896	102071	811532	3353870	56998	72981
**Dak84_RNA_U251**	46157798	271003	160344	1267963	29684	7563374	4726190	10362613	435226	4953805	4063418	14926	35663
**American WT_Ribo_Vero**	42920457	22724492	106204	1037987	493	14454387	49788	281217	982	478651	142016	96	227
**American WT_RNA_Vero**	43613503	1612510	187467	856230	160488	8181954	5415261	15609537	10537	4053875	2552102	1068	1819
**uORF1-KO_Ribo_Vero**	41997673	22189415	109004	1446320	168	14483758	75542	395398	1423	634978	162038	171	413
**uORF1-KO_RNA_Vero**	47048004	1803054	257136	1097563	153110	8808847	6409513	16944062	11427	4448707	2741891	384	727
**uORF2-PTC1_Ribo_Vero**	46074463	32930505	82004	1326289	468	7889051	37898	287468	515	319894	83413	58	128

**uORF2-PTC1_RNA_Vero**	46265642	1698862	158444	1993840	411069	7418461	6052920	17267630	10284	4324269	2666466	645	1023
**African-like_Ribo_Vero**	43304691	33085249	109081	775645	1168	5499218	32731	244637	723	331020	68134	53	115
**African-like_RNA_Vero**	49766329	1810096	173096	1805806	485750	7807053	6188170	18616880	12958	4943514	3073040	453	946
**PE243_Ribo_C6/36_CHX_REP1**	11816810	6036119	266568	7514	374	668215	19023	496758	916	296197	231370	2533	7121
**PE243_RNA_C6/36_CHX_REP1**	39609324	14445762	17257	36511	1199	5234521	339784	5990388	608	5575595	3852567	250	759
**PE243_Ribo_C6/36_CHX_REP2**	16116140	10812188	135957	6681	251	663580	9678	1061821	561	297515	155713	3771	10088
**PE243_RNA_C6/36_CHX_REP2**	31795712	8642445	13577	33503	1134	4562398	404576	5845569	579	5147346	3424889	227	509

Supp Table 10. Genbank accession numbers for rRNA

	**rRNA accession numbers**
Chlorocebus sabaeus	NR_003287.2, NR_023379.1, NR_003285.2, NR_003286.2, AY603036.1, AF420058.1, AF420040.1, AY633510.1, AF352382.1, L35185.1, DQ983926.1, KJ193255.1, M30951.1, M30950.1, M30952.1, KJ193272.1, KJ193259.1, KJ193258.1, KJ193256.1, KJ193255.1, KJ193045.1, KJ193042.1, KJ193044.1, KJ193041.1, KJ193019.1, KJ193018.1, KJ193017.1, AF420040.1
Homo sapiens	NR_003287.4, NR_023379.1, NR_003285.3 and NR_003286.4
Aedes albopictus	L22060, DQ397934.1, DQ397935.1, JX522172.1, AB085210.1, X57172.1, XR_003895909.1

Supp Table 11. Genbank accession numbers for mycoplasma sequences

NC_000908, NC_000912, NC_002771, NC_004432, NC_004829, NC_005364, NC_006360, NC_006908, NC_007294, NC_007295, NC_007332, NC_007633, NC_009497, NC_011025, NC_012806,NC_013511, NC_013948, NC_014014, NC_014448, NC_014552, NC_014751, NC_014760, NC_014921, NC_014970, NC_015153, NC_015155, NC_015431, NC_015725, NC_015946, NC_016638, NC_016807, NC_016829, NC_017502, NC_017503, NC_017504, NC_017509, NC_017519, NC_017520, NC_017521, NC_018077, NC_018149, NC_018406, NC_018407, NC_018408, NC_018409, NC_018410, NC_018411, NC_018412, NC_018413, NC_018495, NC_018496, NC_018497, NC_018498, NC_019552, NC_019949, NC_020076, NC_021002, NC_021007, NC_021025, NC_021083, NC_021283, NC_021831, NC_022575, NC_022807, NC_023062, NC_000912, NC_002771, NC_004432, NC_006908, NC_006360, NC_007295, NC_007294, NC_007332, NC_007633, NC_000908, NC_005364, NC_009497, NC_011025, NC_012806, NC_013511, NC_013948, NC_014014, NC_004829, NC_014448, NC_014552, NC_014751, NC_014760, NC_014921, NC_014970, NC_015155, NC_015153, NC_015431, NC_015725, NC_015946, NC_016638, NC_016807, NC_016829, NC_017502, NC_017503, NC_017504, NC_017509, NC_017519, NC_017520, NC_017521, NC_018077, NC_018149, NC_018406, NC_018407, NC_018408, NC_018409, NC_018410, NC_018411, NC_018412, NC_018413, NC_018495, NC_018496, NC_018497, NC_018498, NC_019552, NC_019949, NC_021002, NC_021007, NC_021025, NC_021083, NC_021283, NC_021831, NC_022575, NC_022807, NC_023062, NC_020076

Supp Table 12. Designated regions to calculate the number of reads per phase

Virus	Region of interest	Coordinates barcharts
PE243	uORF1	25-79
	uORF2	80-107
	Main ORF (Non-overlapping region)	311-480
Dak84	African uORF	25-106
	Main ORF (Non-overlapping region)	311-480
Mutant viruses (American WT, uORF1-KO, uORF2-PTC1)	uORF1	25-79
	uORF2	80-107
	Main ORF (Overlapping region with uORF2 only)	120-310
Mutant virus (African-like)	African uORF	25-106
	Main ORF (Overlapping region with African uORF only)	119-309

Supplementary references

1. Hill CH, Brierley I. (2023). Structural and functional insights into viral programmed ribosomal frameshifting. Annual Rev. Virol. 10:217-242.

2. Stewart, H., Olspert, A., Butt, B.G., and Firth, A.E. (2019). Propensity of a picornavirus polymerase to slip on potyvirus-derived transcriptional slippage sites. Journal of General Virology *100*, 199–205. 10.1099/jgv.0.001189.

3. Ratinier, M., Boulant, S., Combet, C., Targett-Adams, P., McLauchlan, J., and Lavergne, J.-P. (2008). Transcriptional slippage prompts recoding in alternate reading frames in the hepatitis C virus (HCV) core sequence from strain HCV-1. J Gen Virol *89*, 1569–1578. 10.1099/vir.0.83614-0.

4. Irigoyen, N. *et al.* High-Resolution Analysis of Coronavirus Gene Expression by RNA Sequencing and Ribosome Profiling. *PLoS Pathog* **12**, (2016).

5. Rizvi, V. A., Sarkar, M. & Roy, R. Translation regulation of Japanese encephalitis virus revealed by ribosome profiling. *bioRxiv, https://doi.org/10.1101/2020.07.16.206920* (2020).
