# Supplementary material for: Zika viruses encode 5′ upstream open reading frames affecting infection of human brain cells
Source: Nat Commun. 2024 Oct 12;15:8822. doi: 10.1038/s41467-024-53085-9 (PMC11470053; doi:10.1038/s41467-024-53085-9)
Supplement: Supplementary file 3 — Reporting Summary [file 41467_2024_53085_MOESM3_ESM.pdf]

nature portfolio

Corresponding author(s): Dr Nerea Irigoyen

Last updated by author(s): Sep 4, 2024

Reporting Summary

Nature Portfolio wishes to improve the reproducibility of the work that we publish. This form provides structure for consistency and transparency in reporting. For further information on Nature Portfolio policies, see our Editorial Policies and the Editorial Policy Checklist.

Statistics

For all statistical analyses, confirm that the following items are present in the figure legend, table legend, main text, or Methods section.

n/a	Confirmed	
☐	☒	The exact sample size (*n*) for each experimental group/condition, given as a discrete number and unit of measurement
☐	☒	A statement on whether measurements were taken from distinct samples or whether the same sample was measured repeatedly
☐	☒	The statistical test(s) used AND whether they are one- or two-sided<br>*Only common tests should be described solely by name; describe more complex techniques in the Methods section.*
☐	☒	A description of all covariates tested
☐	☒	A description of any assumptions or corrections, such as tests of normality and adjustment for multiple comparisons
☐	☒	A full description of the statistical parameters including central tendency (e.g. means) or other basic estimates (e.g. regression coefficient) AND variation (e.g. standard deviation) or associated estimates of uncertainty (e.g. confidence intervals)
☐	☒	For null hypothesis testing, the test statistic (e.g. *F*, *t*, *r*) with confidence intervals, effect sizes, degrees of freedom and *P* value noted<br>*Give P values as exact values whenever suitable.*
☒	☐	For Bayesian analysis, information on the choice of priors and Markov chain Monte Carlo settings
☒	☐	For hierarchical and complex designs, identification of the appropriate level for tests and full reporting of outcomes
☒	☐	Estimates of effect sizes (e.g. Cohen's *d*, Pearson's *r*), indicating how they were calculated

Our web collection on statistics for biologists contains articles on many of the points above.

Software and code

Policy information about availability of computer code

Data collection

Amplicon libraries were deep-sequenced using an Illumina NextSeq500 platform.
All confocal images were captured with a Zeiss LSM 700 laser scanning microscope with the ZEN microscope software. Image J 1.53t was used to make representative Z-stack montages and merged Z-stack images.

Data analysis

Computational analyses of sequence data

Reads were demultiplexed. Adaptors were trimmed off using the FASTX-Toolkit version 0.0.14 (http://hannonlab.cshl.edu/fastx_toolkit/), and sequences shorter than 25 nt after trimming were discarded. Sequences not linked to an adaptor and sequences constituted of adaptors ligated together were also removed. Reads remaining after this initial selection were sequentially mapped to (i) ribosomal RNA (rRNA); (ii) the American or African ZIKV genomic RNA (viral genome sequences were confirmed by de novo assembly using Trinity version2.8.5); (iii) messenger mRNA (mRNA); (iv) non-coding RNA (ncRNA); (v) genomic DNA (gDNA) and (vi) a contaminants database, using Bowtie (version 1.2.3) with parameters -v n_mismatches --best (i.e., maximum of n_mismatches mismatches permitted, report best match). The remaining reads were classified as unmapped.

The rRNA databases included the Genbank accession numbers are listed in Supp Table 10. The mRNA sequences for each host were downloaded from NCBI RefSeq. The ncRNA databases for C. sabaeus were retrieved from Ensembl release 91 and compiled with 534 tRNA sequences of M. mulatta and Genbank accession XM_007980346; for H. sapiens, the ncRNA sequences were downloaded from Ensembl release 105 and for A. albopictus from Ensembl release 55. The gDNA sequences for each host were retrieved from the same Ensembl releases as the ncRNA. The contaminants database comprises Genbank accessions of potential contaminants that might be found in the lab and are used as quality control to ensure that no/very few reads map to this database. The database includes viruses (e.g., HSV-1, PRRSV) and bacteria (e.g., E. coli) that are regularly used within the Division of Virology, University of Cambridge, and other common potential

contaminants (e.g., mycoplasma sequences).

For manuscripts utilizing custom algorithms or software that are central to the research but not yet described in published literature, software must be made available to editors and reviewers. We strongly encourage code deposition in a community repository (e.g. GitHub). See the Nature Portfolio guidelines for submitting code & software for further information.

Data

Policy information about availability of data

All manuscripts must include a data availability statement. This statement should provide the following information, where applicable:

- Accession codes, unique identifiers, or web links for publicly available datasets
- A description of any restrictions on data availability
- For clinical datasets or third party data, please ensure that the statement adheres to our policy

All data are available in the main text, the Supplementary Information and the Figshare repository (https://doi.org/10.6084/m9.figshare.25431730).

Ribo-Seq and RNA-Seq sequencing data have been deposited in the ArrayExpress database (http://www.ebi.ac.uk/arrayexpress) under the accession codes: E-MTAB-5418 (Ribo-Seq and RNA-Seq in Vero and C6/36 cells infected with PE243 (MOI:3) and pretreated with CHX; https://www.ebi.ac.uk/biostudies/arrayexpress/studies/E-MTAB-5418?query=E-MTAB-5418), E-MTAB-12967 (RNA-Seq in Vero and U251 cells infected with PE243 (MOI:3) and Dak84 (MOI:3) and flash-frozen; https://www.ebi.ac.uk/biostudies/arrayexpress/studies/E-MTAB-12967?query=E-MTAB-12967), E-MTAB-12968 (RNA-Seq in Vero cells infected with American WT, African-like, uORF1-KO and uORF2-PTC1 (MOI:3); (https://www.ebi.ac.uk/biostudies/arrayexpress/studies/E-MTAB-12968?query=E-MTAB-12968), E-MTAB-12969 (Ribo-Seq in Vero cells infected with American WT, African-like, uORF1-KO and uORF2-PTC1 (MOI:3); https://www.ebi.ac.uk/biostudies/arrayexpress/studies/E-MTAB-12969?query=E-MTAB-12969) and E-MTAB-12970 (Ribo-Seq in Vero and U251 cells infected with PE243 (MOI:3) and Dak84 (MOI:3) and flash-frozen; https://www.ebi.ac.uk/biostudies/arrayexpress/studies/E-MTAB-12970?query=E-MTAB-12970).

Research involving human participants, their data, or biological material

Policy information about studies with human participants or human data. See also policy information about sex, gender (identity/presentation), and sexual orientation and race, ethnicity and racism.

Reporting on sex and gender	*Use the terms sex (biological attribute) and gender (shaped by social and cultural circumstances) carefully in order to avoid confusing both terms. Indicate if findings apply to only one sex or gender; describe whether sex and gender were considered in study design; whether sex and/or gender was determined based on self-reporting or assigned and methods used. Provide in the source data disaggregated sex and gender data, where this information has been collected, and if consent has been obtained for sharing of individual-level data; provide overall numbers in this Reporting Summary. Please state if this information has not been collected. Report sex- and gender-based analyses where performed, justify reasons for lack of sex- and gender-based analysis.*
Reporting on race, ethnicity, or other socially relevant groupings	*Please specify the socially constructed or socially relevant categorization variable(s) used in your manuscript and explain why they were used. Please note that such variables should not be used as proxies for other socially constructed/relevant variables (for example, race or ethnicity should not be used as a proxy for socioeconomic status). Provide clear definitions of the relevant terms used, how they were provided (by the participants/respondents, the researchers, or third parties), and the method(s) used to classify people into the different categories (e.g. self-report, census or administrative data, social media data, etc.) Please provide details about how you controlled for confounding variables in your analyses.*
Population characteristics	*Describe the covariate-relevant population characteristics of the human research participants (e.g. age, genotypic information, past and current diagnosis and treatment categories). If you filled out the behavioural & social sciences study design questions and have nothing to add here, write "See above."*
Recruitment	*Describe how participants were recruited. Outline any potential self-selection bias or other biases that may be present and how these are likely to impact results.*
Ethics oversight	*Identify the organization(s) that approved the study protocol.*

Note that full information on the approval of the study protocol must also be provided in the manuscript.

Field-specific reporting

Please select the one below that is the best fit for your research. If you are not sure, read the appropriate sections before making your selection.

[x] Life sciences [ ] Behavioural & social sciences [ ] Ecological, evolutionary & environmental sciences

For a reference copy of the document with all sections, see nature.com/documents/nr-reporting-summary-flat.pdf

Life sciences study design

All studies must disclose on these points even when the disclosure is negative.

Sample size	Sample sizes for female mosquito populations were chosen based on previous published literature (10.1038/s41467-021-21199-z), between 10 and 20 individuals per time point per Zika virus mutant.

No a priori sample-size calculation was performed for sample sizes for imaging (Figs. 6B, 6C and 7C-G) but the number of samples were high enough to be statistical significant (i.e., 30 positive cells per mutant virus in three independent biological replicates for Fig. 6B and 6C and 33 images with at least 400 nuclei for Figs. 7C-G) from four ALI-COs slices derived from two independent cortical organoids.

Data exclusions	Data excluded from the analyses (i.e., stratification per read length in Ribo-Seq analyses) have been appropriately justified in the text.
Replication	Robustness of experimental findings was supported by obtaining consistent results across multiple experiments.<br>Experimental data was collected from at least three independent biological replicates unless stated in the text. All attempts at replication were successful. Ribo-Seq and RNA-Seq data were collected from only two independent biological replicate as previously published (10.1371/journal.ppat.1005473) but results were reproducible as they were replicated between different cell lines.
Randomization	ALI-COs and mosquitoes were randomly allocated to experimental groups.
Blinding	Investigators were blinded during confocal data collection.<br>Blinding was unnecessary for the rest of experiments because the biological measurements (e.g., virus quantification) were objective and thus unlikely to be influenced by the experimenter.

Reporting for specific materials, systems and methods

We require information from authors about some types of materials, experimental systems and methods used in many studies. Here, indicate whether each material, system or method listed is relevant to your study. If you are not sure if a list item applies to your research, read the appropriate section before selecting a response.

Materials & experimental systems

n/a	Involved in the study	
☐	☒	Antibodies
☐	☒	Eukaryotic cell lines
☒	☐	Palaeontology and archaeology
☐	☒	Animals and other organisms
☒	☐	Clinical data
☐	☒	Dual use research of concern
☒	☐	Plants

Methods

n/a	Involved in the study	
☒	☐	ChIP-seq
☒	☐	Flow cytometry
☒	☐	MRI-based neuroimaging

Antibodies

Antibodies used

For immunoblotting:

Primary Antibodies:
Anti-GAPDH: mouse IgM, Sigma-Aldrich (G8795) Dilution 1:20,000: https://www.sigmaaldrich.com/GB/en/product/sigma/g8795
Anti-FLAG: mouse IgG, Sigma-Aldrich (SAB4301135) Dilution 1:2,000: https://www.sigmaaldrich.com/GB/en/substance/antimouseiggfabspecificantibodyproducedingoat1234598765
Anti-E protein: rabbit IgG, GeneTex (GTX133314) Dilution 1:1,000: https://www.genetex.com/Product/Detail/Zika-virus-Envelope-protein-antibody/GTX133314
Anti-Lamin A+C: rabbit IgG Abcam (ab108922) Dilution: 1:1,000: https://www.abcam.com/en-us/products/primary-antibodies/lamin-a-lamin-b1-lamin-c-antibody-epr4068-ab108922
Anti-H2A: rabbit IgG Abcam (ab1777308) Dilution: 1:1,000: https://www.abcam.com/en-us/products/primary-antibodies/histone-h2a-antibody-epr17470-chip-grade-ab177308
Anti-Vimentin: mouse IgG1 Abcam (ab8069) Dilution 1:1,000: https://www.abcam.com/en-us/products/primary-antibodies/vimentin-antibody-v9-cytoskeleton-marker-ab8069
Anti-ERp72: rabbit IgG Cell Signaling (5033) Dilution 1:1,000: https://www.cellsignal.com/products/primary-antibodies/erp72-d70d12-xp-rabbit-mab/5033
Anti-eIF2alpha: rabbit IgG Cell Signaling (9722) Dilution 1:1,000: https://www.cellsignal.com/products/primary-antibodies/eif2a-antibody/9722
Anti-p-eIF2alpha: rabbit IgG Cell Signaling (9721) Dilution 1:1,000: https://www.cellsignal.com/products/primary-antibodies/phospho-eif2a-ser51-antibody/9721
Anti-mCherry: rabbit IgG Abcam (ab167453) Dilution 1:1,000: https://www.abcam.com/en-us/products/primary-antibodies/mcherry-antibody-ab167453

Secondary Antibodies:
Anti-Rabbit Goat 800 Licor, IRDye 926-32211 Dilution 1:1,000
Anti-Mouse IgM Donkey 680 Licor, IRDye 926-68180 Dilution 1:1,000
Anti-Mouse Goat 800 Licor, IRDye 926-32210 Dilution 1:1,000

For immunofluorescence:

	Primary Antibodies:<br>Anti-E protein: rabbit IgG, GeneTex (GTX133314) Dilution 1:200: https://www.genetex.com/Product/Detail/Zika-virus-Envelope-protein-antibody/GTX133314<br>Anti-flavivirus Ag group: mouse IgG, Merck (D1-4G2-4-15) Dilution 1:200: https://www.merckmillipore.com/GB/en/product/Anti-Flavivirus-Group-Antigen-Antibody-clone-D1-4G2-4-15,MM_NF-MAB10216-I-100UG?ReferrerURL=https%3A%2F%2Fwww.google.com%2F<br>Anti-flavivirus Ag group: rabbit IgG, Absolute antibody (Ab00230-23.0) Dilution 1:200: https://absoluteantibody.com/product/Anti-Flavivirus-group-antigen-D1-4G2-4-15-4G2/Ab00230-23.0_Rabbit_IgG/<br>Anti-FLAG: mouse IgG, Sigma-Aldrich (SAB4301135) Dilution 1:500: https://www.sigmaaldrich.com/GB/en/substance/antimouseiggfabspecificantibodyproducedingoat1234598765<br>Anti-FLAG: rabbit IgG, Merck (F7425) Dilution 1:100: https://www.sigmaaldrich.com/GB/en/product/sigma/f7425<br>Anti-Vimentin: mouse IgG1, Abcam (ab8069) Dilution 1:500: https://www.abcam.com/en-us/products/primary-antibodies/vimentin-antibody-v9-cytoskeleton-marker-ab8069<br>Anti-Actin: mouse IgG, Proteintech (66009) Dilution 1:500: https://www.ptglab.com/products/Pan-Actin-Antibody-66009-1-Ig.htm<br>Anti-Tubulin: rat, hybridoma. Kind gift from Dr Colin Crump (University of Cambridge) Dilution 1:10<br>Anti-MAP2: goat IgG, antibodies.com (A104327) Dilution 1:1,000: https://www.antibodies.com/map2-antibody-a104327<br>Anti-Nestin: mouse IgG1, Abcam (ab22035) Dilution 1:500: https://www.abcam.com/en-us/products/primary-antibodies/nestin-antibody-10c2-neural-stem-cell-marker-ab22035<br>Anti-GFAP: rabbit IgG, antibodies.com (A85419) Dilution 1:1,000: https://www.antibodies.com/gfap-antibody-a85419<br><br>Secondary Antibodies:<br>Anti-Rabbit Goat 488 Invitrogen Alexa Fluor (A11008) Dilution 1:1,000<br>Anti-Rabbit Donkey 488 Abcam (ab150073) Dilution 1:1,000<br>Anti-Mouse Goat 488 Invitrogen Alexa Fluor (A11001) Dilution 1:1,000<br>Anti- Rabbit Donkey 594 Invitrogen Alexa Fluor (A21207) Dilution 1:1,000<br>Anti-Mouse Donkey 594 Invitrogen Alexa Fluor (A21203) Dilution 1:1,000<br>Anti-Rat Goat 568 Invitrogen Alexa Fluor (A11077) Dilution 1:1,000
Validation	Each primary antibody has been validated according to the manufacturer's database and with the data provided in the manuscript.<br>Anti-Tubulin provided by Dr Colin Crump was validated in this publication (10.1128/JVI.00595-19).

Eukaryotic cell lines

Policy information about cell lines and Sex and Gender in Research

Cell line source(s)	African green monkey cells: Vero cells (ATCC, CCL81)<br>Human glioblastoma-astrocytoma cells: U-251 cells (ATCC, CRL-1620)<br>Aedes albopictus: C6/36 cells (ATCC, CLR 1660)<br>Human induced pluripotent stem cell-derived glutamatergic cortical neuros (i3Neurons) supplied by the Deane Lab were originally provided by Michael Ward's lab (NINDS/NIH, USA).<br>Air-liquid interface cerebral organoids (ALI-COs) were generated from the embryonic stem cell H9 line (WiCell) and supplied by the Lakatos Lab.
Authentication	Vero, U-251 and C6/36 cells were obtained from the ATCC.<br>i3Neurons were authenticated as described in Wang et al., 2017 (10.1016/j.stemcr.2017.08.019).<br>ALI-COs were authenticated as described in Szebényi et al., 2021 (10.1038/s41593-021-00923-4) and Giandomenico et al., 2019 (10.1038/s41593-019-0350-2).
Mycoplasma contamination	All cell lines tested negative for mycoplasma contamination.
Commonly misidentified lines<br>(See ICLAC register)	*Name any commonly misidentified cell lines used in the study and provide a rationale for their use.*

Animals and other research organisms

Policy information about studies involving animals; ARRIVE guidelines recommended for reporting animal research, and Sex and Gender in Research

Laboratory animals	All in vivo mosquito experiments used the 15th and 16th laboratory generations of an Aedes (Ae.) aegypti colony originally established from wild specimens caught in Barranquilla, Colombia, in 2017. Mosquitoes were maintained under controlled insectary conditions (28°±1°C, 12h:12h light: dark cycle and 70% relative humidity). Larvae were reared in dechlorinated tap water supplemented with a standard diet of TetraMin fish food (Tetra). Adults were kept in insect cages (BugDorm) with permanent access to a 10% sucrose solution.
Wild animals	The study did not involve wild-animals.
Reporting on sex	Only female mosquitoes were used as they are the ones susceptible to transmit Zika virus.
Field-collected samples	The study did not involve samples field-collected samples.

Ethics oversight	No ethical approval was required. Invertebrate animals do not fall under any regulation for experimentation.<br>The rabbit blood used to make artificial blood meals is from a commercial vendor and therefore does not require ethical clearance.

Note that full information on the approval of the study protocol must also be provided in the manuscript.

Plants

Seed stocks	*Report on the source of all seed stocks or other plant material used. If applicable, state the seed stock centre and catalogue number. If plant specimens were collected from the field, describe the collection location, date and sampling procedures.*
Novel plant genotypes	*Describe the methods by which all novel plant genotypes were produced. This includes those generated by transgenic approaches, gene editing, chemical/radiation-based mutagenesis and hybridization. For transgenic lines, describe the transformation method, the number of independent lines analyzed and the generation upon which experiments were performed. For gene-edited lines, describe the editor used, the endogenous sequence targeted for editing, the targeting guide RNA sequence (if applicable) and how the editor was applied.*
Authentication	*Describe any authentication procedures for each seed stock used or novel genotype generated. Describe any experiments used to assess the effect of a mutation and, where applicable, how potential secondary effects (e.g. second site T-DNA insertions, mosiacism, off-target gene editing) were examined.*
